# Microbiota-driven tryptophan metabolism and AhR triggered intestinal stem cell differentiation: mechanisms of huangqin decoction in ulcerative colitis repair

**DOI:** 10.1186/s13020-025-01302-y

**Published:** 2026-01-13

**Authors:** Roude Li, Xiaoxia Liao, Xin Fu, Xiaoxin Li, Xiyi Liao, Shuimei Cen, Jiayang Zeng, Longyun Huang, Honggang Chi, Ying Zou

**Affiliations:** 1 The First Dongguan Affiliated Hospital, Guangdong Medical University, Dongguan, 523710 China; 2 The Second School of Clinical Medicine, Guangdong Medical University, Dongguan, 523000 China; 3 Suixi County People’s Hospital the Affiliated Hospital of Guangdong Medical University, Zhanjiang, 524300 China; 4 Dongguan Guancheng Hospital, Dongguan, 523000 China; 5 Department of Traditional Chinese Medicine, Dongguan Liaobu Hospital, Dongguan, 523000 China

**Keywords:** Ulcerative colitis, Huangqin decoction, Intestinal stem cell, AhR, Tryptophan metabolite, Gut microbiota

## Abstract

**Background:**

Promoting intestinal barrier repair and epithelial regeneration is a core therapeutic objective in managing ulcerative colitis (UC). Intestinal stem cell (ISC) differentiation is pivotal in sustaining epithelial renewal and mucosal homeostasis. Huangqin decoction (HQD), a classical herbal formulation comprising *Scutellaria baicalensis, Ziziphus jujuba, Paeonia lactiflora,* and *Glycyrrhiza uralensis,* is clinically used for inflammatory bowel disease. Nevertheless, how HQD precisely regulates ISC differentiation to promote UC repair remains unclear.

**Purpose:**

This research sought to assess whether HQD ameliorates UC by concurrently modulating the gut microbiome, tryptophan metabolism, aryl hydrocarbon receptor (AhR) activation, and ISC differentiation.

**Methods:**

Mice developed colitis after drinking water with a 3.5% (w/v) concentration of dextran sulfate sodium. We evaluated HQD effects on colon length, weight trajectory, disease activity index score, histological damage, and colonic inflammatory mediator abundance. Metagenomic sequencing resolved microbiota restructuring, while UPLC-MS/MS quantified fecal tryptophan metabolites such as indole derivatives. AhR pathway activity (AhR, CYP1A1), its downstream cytokine IL-22, and ISC fate were mapped by combining immunofluorescence, ELISA, Western blot, and RT-qPCR, probing Lgr5 for stem-cell identity and MUC2, LYZ, and ChgA for lineage-specific differentiation. The involvement of AhR and gut microbiota was investigated using AhR inhibitors and broad-spectrum antibiotics.

**Results:**

High-dose HQD significantly alleviated colitis symptoms, reduced colon damage, and corrected gut dysbiosis. HQD increased the abundance of related bacteria that elevated colonic levels of indole-3-propionic acid, indole-3-acetamide, and tryptamine, acting as AhR ligands that upregulate AhR and its downstream targets CYP1A1 and IL-22. Crucially, HQD promoted a shift in expression from the ISC marker Lgr5 toward differentiation markers MUC2, LYZ, and ChgA, indicating enhanced ISC differentiation and improved barrier function. These effects were effectively blocked by AhR inhibition or antibiotic treatment.

**Conclusion:**

HQD restores intestinal mucosal integrity and attenuates colonic inflammation by modulating gut microbiota composition, increasing microbial tryptophan metabolites with AhR-agonist activity, activating the AhR signaling pathway, and promoting ISC differentiation into functional epithelial cells. This work reveals a novel “microbiota–tryptophan metabolism–AhR–ISC differentiation” axis underlying HQD’s therapeutic efficacy in UC.

**Supplementary Information:**

The online version contains supplementary material available at 10.1186/s13020-025-01302-y.

## Introduction

Characterized by ongoing and recurrent inflammation of the gastrointestinal tract, inflammatory bowel disease (IBD) encompasses ulcerative colitis (UC) and Crohn’s disease. UC appears as a gradually worsening inflammatory disease of the colonic mucosa, featuring epithelial ulceration, crypt abscess formation, and impaired barrier function. Its pathogenesis involves genetic susceptibility, dysregulated immune responses (particularly elevated pro-inflammatory cytokines), compromised epithelial integrity, environmental triggers, etc. [1, 2]. Owing to this multifactorial etiology, effective long-term therapeutic strategies remain limited.

The human gut harbors a diverse microbial ecosystem that co-evolved with the host to establish mutualistic symbiosis [3]. Substantial evidence points to gut microbiota dysbiosis as a pivotal factor in the development of UC. Disruption of microbial homeostasis—characterized by reduced species diversity, altered abundance, and an aberrant *Firmicutes/Bacteroidetes* ratio—compromises intestinal barrier function, facilitating bacterial translocation and aberrant immune activation. Consequently, this dysbiosis perpetuates mucosal inflammation and disease progression.

Microbiota-derived metabolites are critical mediators of intestinal barrier integrity [4]. Key metabolites such as short-chain fatty acids, bile acids, and tryptophan breakdown products function as signaling molecules and metabolic substrates that modulate inflammatory pathways [5]. Notably, current evidence indicates a reduction in aryl hydrocarbon receptor (AhR) expression within the intestinal tissue of IBD patients; at the same time, the mucosal barrier is restored in mouse models of intestinal inflammation through AhR signaling via IL-22 [6]. Gut bacteria metabolize tryptophan into indole derivatives that activate AhR. The tryptophan-AhR axis drives Intestinal stem cell (ISC) proliferation, promotes mucosal repair, and suppresses inflammation in experimental colitis [7], representing a promising therapeutic target.

Emerging research indicates that microbiota dysbiosis and disrupted tryptophan metabolism impair the renewal and differentiation of ISC—processes essential for epithelial regeneration and barrier restoration [8, 9]. Achieving mucosal healing is the primary treatment goal in UC [10], which fundamentally relies on functional ISC. These cells give rise to all epithelial lineages of the intestine, encompassing enterocytes, goblet cells, enteroendocrine cells, and Paneth cells [11]. Elucidating how microenvironmental cues regulate ISC behavior is thus crucial for developing regenerative UC therapies.

Traditional Chinese medicine (TCM) has shown effectiveness in UC management, offering advantages such as sustained remission and favorable safety profiles. Huangqin decoction (HQD), a classic TCM formulation, mitigates dextran sulfate sodium (DSS)-evoked murine colitis through restructuring gut microbiota composition, activating the mTOR pathway, and suppressing the NF-κB pathway [12, 13]. Although prior studies indicate that HQD alleviates colitis symptoms by modulating gut microbiota and that AhR signaling is critically involved in ISC regulation, whether HQD ameliorates colitis through the microbiota–tryptophan metabolism–AhR–ISC axis remains to be elucidated.

This research implemented a murine acute colitis model using 3.5% DSS to validate HQD’s dose-dependent efficacy and therapeutic effects. Metagenomics and targeted tryptophan metabolomics revealed HQD-modulated alterations in gut microbiota and tryptophan metabolites. Subsequent in vivo experiments demonstrated that HQD activates AhR via these pathways to drive ISC differentiation and restore the mucus barrier. Finally, AhR inhibitors and broad-spectrum antibiotics confirmed the essential roles of AhR signaling and gut microbiota in this mechanism. Thus, we propose a potential novel therapeutic strategy by elucidating how HQD promotes epithelial regeneration and repair by influencing the gut microbiota-tryptophan-AhR-ISC axis in ulcerative colitis.

## Materials and methods

### Materials

The crude constituents of HQD were procured from Kangmei Pharmaceutical Co., Ltd. (Guangdong, China). SPF Biotechnology Co., Ltd. (Beijing, China) supplied the 7-week-old male C57BL/6 mice. Macklin Biochemical Technology Co., Ltd. (Shanghai, China) provided the DSS. The primers were sourced from Sangon Biotech (Shanghai, China). AhR inhibitor CH223191 (A8609) was purchased from APExBIO Technology LLC (USA). Antibiotic cocktail includes Metronidazole (M813526), Cefoxitin (C859229), Vancomycin hydrochloride (V6062), and Gentamycin Sulfate (G6064), which were procured from Macklin Biochemical Technology Co., Ltd. (Shanghai, China). The enzyme-linked immunosorbent assay (ELISA) kit of IL-22 (E-EL-M2446) was purchased from Elabscience (Wuhan, China). The antibodies used in the experiment were as follows: Lgr5 (ab75850, clone: EPR3065Y, Abcam, UK), ChgA (10529-1-AP, Proteintech, China), LYZ (15013-1-AP, Proteintech, China), AhR (67785-1-Ig, clone: 2D1F9, Proteintech, China), CYP1A1 (sc-25304, clone: B-4, Santa Cruz Biotechnology, USA), E-Cadherin (BF0219, clone: AFfirm06(AFB1836), Affinity Biosciences, China), GAPDH (60004-1-Ig, clone: 1E6D9, Proteintech, China), and β-actin (AF7018, Affinity Biosciences, China).

### Preparation and composition of HQD

HQD was prepared by decocting a blend of *Scutellaria baicalensis Georgi*, *Paeonia lactiflora Pall.*, *Glycyrrhiza uralensis Fisch.*, and *Ziziphus jujube Mill*. (3:2:2:2 ratio) in pure water through two 30-min extractions. The filtrates were combined, concentrated to 1 g/mL, and refrigerated at 4 °C. Samples were thawed and centrifuged (13,800 × *g*, 4 °C, 15 min). A 300 μL supernatant was mixed with 1000 μL of extraction solvent, made up of methanol, acetonitrile, and water in a 2:2:1 ratio by volume, with isotope-labeled internal standards included. After vortexing (30 s) and ice-bath sonication (5 min), mixtures were kept at − 20 °C (1 h), then spun down again at 13,800 × *g* and 4 °C for 15 min. The supernatant was filtered (0.22 μm) into vials.

A Vanquish UPLC system with a Kinetex C18 column (2.1 × 100 mm, 2.6 μm) was utilized for chromatography. Mobile phase: (A) 0.01% acetic acid/water, (B) isopropanol: acetonitrile (1:1, v/v); injection volume 2 μL at 4 °C. Orbitrap Exploris 120 MS operated in DDA mode (Xcalibur v4.4): sheath/aux gas 50/15 arb, capillary 320 °C, Full resolution 60 K, MS1/MS2 resolution 15 k, collision energy SNCE 20/30/40, spray voltage ± 3.8/− 3.4 kV.

### Animals and experimental protocal

Following ethical guidelines for animal use, eighty 7-week-old male C57BL/6 J mice, weighing about 22 g each, were maintained in a pathogen-free environment with proper food and water. After spending a week adjusting, to establish the optimal therapeutic dose of HQD, C57BL/6 J male mice were allocated to five arms randomly (each with N = 6): Normal, DSS, 5-aminosalicylic acid (5-ASA) at 0.52 g/kg, HQD-L at 4.55 g/kg, and HQD-H at 9.1 g/kg. Following identification of the optimal HQD dose (9.1 g/kg), mice were stratified into eight arms (N = 6 each) for mechanistic investigation: Normal group, DSS group, 5-ASA group, HQD group, DSS + CH223191 group (designated group DC), DSS + CH223191 + HQD group (designated group DCH), Antibiotic cocktail (Abx) + DSS group (designated group AD), and Abx + DSS + HQD group (designated group ADH).

Mice in the AD and ADH groups received a broad-spectrum Abx (cefoxitin, gentamicin, metronidazole, and vancomycin at 1, 1, 2, and 1 mg/mL, respectively) ad libitum for two weeks to deplete gut microbiota. After confirming microbial clearance, all groups except the Normal group (maintained on autoclaved water) were administered 3.5% DSS solution ad libitum for 7 days. During DSS exposure, the HQD, DCH, and ADH groups received daily oral gavage of HQD (9.1 g/kg), while the 5-ASA group was treated with 5-ASA (0.52 g/kg). Concurrently, the DC and DCH groups were injected intraperitoneally with the AhR inhibitor CH223191 (10 mg/kg) daily for 7 days. Fecal samples were collected for microbial sequencing and metabolomic analysis. Following a 3-day recovery period with autoclaved water, mice were euthanized, and experimental tissues were harvested. Clinical parameters were recorded daily throughout the modeling and treatment phases, including weight trajectory, coat condition, locomotor activity, and stool consistency. The disease activity index (DAI) was used to assess colitis severity, as outlined in Additional file 1: Table S1.

### Histological assessment

Following experimental termination, colons were excised and measured. After overnight fixation using 4% paraformaldehyde, distal colon segments were embedded in paraffin and cut into 4 μm sections. Hematoxylin–eosin (H&E) staining was applied to consecutive sections to enable evaluation of crypt distortion, immune cell infiltration, and epithelial damage severity histopathologically (detailed in Additional file 1: Table S2). Parallel sections received Alcian blue/periodic acid-Schiff (AB-PAS) staining to quantify goblet cell depletion, a key indicator of intestinal barrier compromise in ulcerative colitis pathogenesis.

### Intestinal permeability assay

Measure intestinal permeability in mice using fluorescein isothiocyanate (FITC)-dextran. After fasting and water deprivation for 4 h, mice were orally administered FITC-dextran (600 mg/kg). Blood was collected from the eye socket after tribromoethanol anesthetization 4 h later, and a Microplate reader was used to measure the fluorescence intensity. Finally, the serum concentration of FITC-dextran was determined with a standard curve.

### Metagenomic analysis

After model establishment, fresh fecal samples from all experimental groups were collected in sterile tubes and submitted to Shanghai Majorbio Bio-Pharm Technology Co., Ltd. for examination. 1% agarose gel electrophoresis was performed on the samples after DNA was extracted. Subsequent procedures included DNA fragmentation, PE library construction, bridge PCR amplification, and Illumina sequencing. Raw sequencing reads underwent demultiplexing, quality trimming, and purification. The processed data were assembled for gene prediction, with predicted genes annotated and classified for taxonomic and functional characterization. Multidirectional statistical analyses were conducted based on these results, followed by visualization.

### Targeted metabolomics analysis

10 μL internal standard (4000 ng/mL), 190 μL methanol: water (4:1, v/v), and fecal samples (25 mg) were homogenized through cryogenic grinding (− 10 ℃, 6 min, 50 Hz). Subsequent extraction involved ultrasonication (5 ℃, 30 min, 40 kHz), static incubation (− 20 ℃, 30 min), and centrifugation (4 ℃, 13,000 *g*, 15 min). Supernatants were nitrogen-dried, reconstituted in 70 μL of 1% acetonitrile/0.1% formic acid, sonicated (15 min), and centrifuged.

LC–ESI–MS/MS quantification utilized an ACQUITY UPLC HSS T3 column (2.1 × 150 mm, 1.8 μm) with formic acid-modified water/acetonitrile gradient (details in Additional file 1: Table S3). A QTRAP 6500 + system operated in positive/negative modes with key parameters: curtain gas 35 psi; ion spray voltage ± 5500/− 4500 V; ion source 550 ℃. Data integration (AB Sciex software) and statistical analysis (Meiji Cloud platform) completed the workflow.

### Immunofluorescence staining

Deparaffinized colon sections were sealed with a blocking solution for 2 h. Overnight incubation of the tissue sections with Lgr5 antibody was done at 4 ℃. The following day, anti-rabbit IgG (FITC) was added, and the sections were incubated for an hour at room temperature in the dark. The tissue was exposed to DAPI at room temperature for 10 min after incubation, and an immunofluorescence quenching agent was used to seal the slices. Finally, the slices were observed using confocal laser scanning microscopy.

### Reverse transcription quantitative polymerase chain reaction (RT-qPCR)

The RNA-easy Isolation Reagent from Vazyme Biotech Co., Ltd. was employed to isolate the total RNA from colon tissues. Synthesized cDNA was quantified, and qPCR was performed using SYBR^®^ Green (Takara Bio Inc.) on a QuantStudio 3 system (Thermo Fisher Scientific). Gene expression levels were normalized to GAPDH and calculated via the 2^−ΔΔCt^ method. Additional file 1: Table S4 contains the detailed sequences of primers.

### Western blotting

The protein was extracted by mashing the colons and cleaving them with RIPA lysate. After adding loading buffer and boiling the samples for five minutes, the denatured proteins were isolated using SDS-PAGE gel electrophoresis. Upon the transfer of protein samples to a PVDF membrane, primary (E-cadherin, AhR, CYP1A1, Lgr5, ChgA, LYZ, β-actin, GAPDH) and secondary antibodies were added and incubated. The blots were created with an ECL Chemiluminescence kit, and the integrated density of pixels in each membrane was calculated using ImageJ software.

### ELISA

Following the manufacturer’s instructions for the ELISA assay kit, ELISA was utilized to assess IL-22 levels in mouse colon tissue.

### Statistical analysis

R software (version 4.1.2) or GraphPad Prism was utilized for all statistical analyses. Statistically significant differences between two groups were analyzed by Student’s t-test; multiple group comparisons were examined by one-way analysis of variance (ANOVA) followed by Tukey’s post hoc test, with statistical significance defined at *P* < 0.05.

## Results

### HQD dose-dependently improved the symptoms in DSS-induced mice

**Fig. 1:**
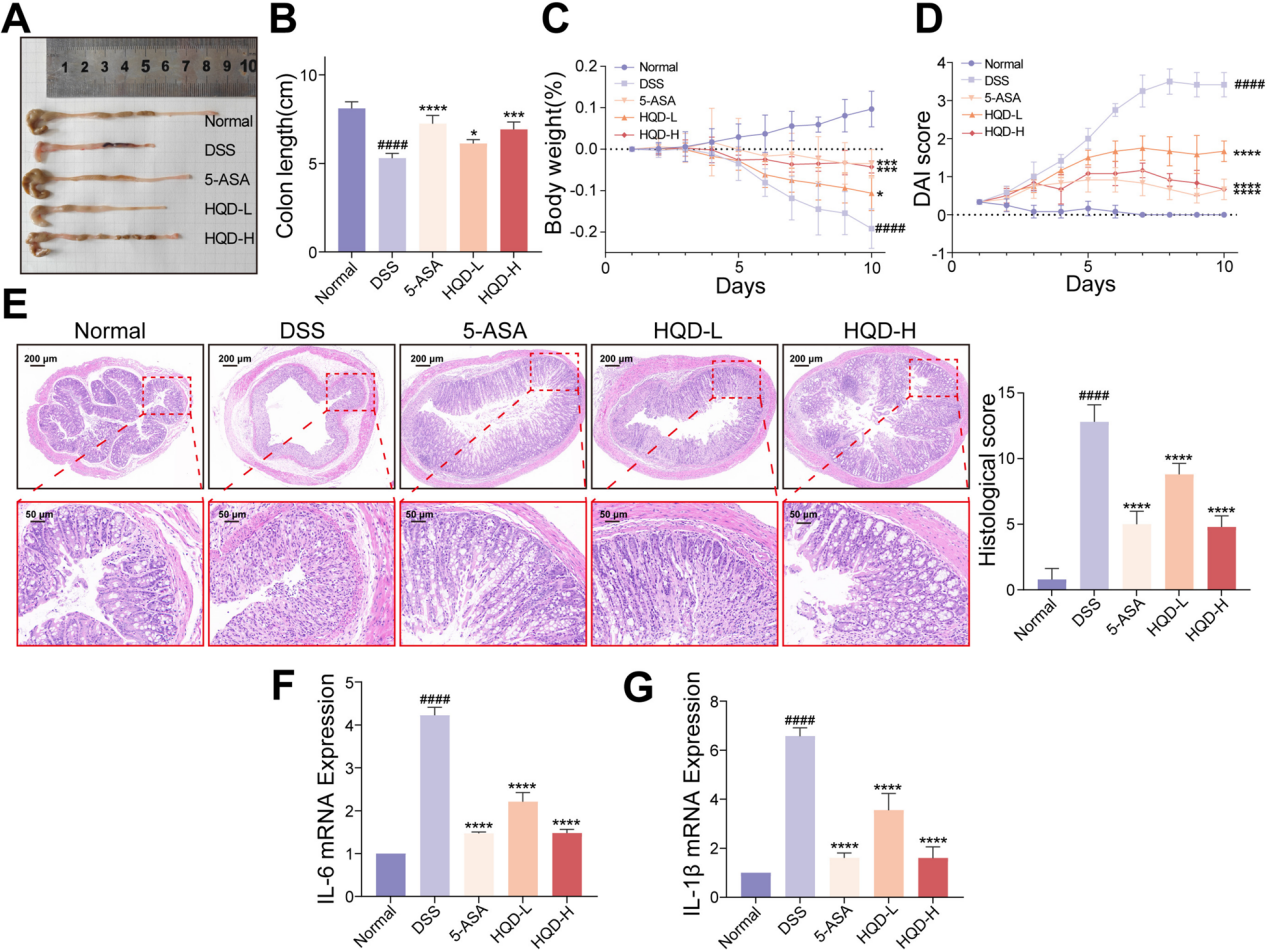
HQD dose-dependently improved the symptoms in DSS-induced mice. **A**, **B** Colon length. **C** Body weight trajectory. **D** The DAI score. **E** H&E staining. **F**, **G** IL-6 and IL-1β mRNA expression levels in colon tissues. Data were presented as mean ± SD (n = 4–6). ^####^*P* < 0.0001, vs Normal group. *P < 0.05, ***P < 0.001, ****P < 0.0001, vs DSS group

The therapeutic potential of HQD in UC was evaluated by treating DSS-induced mice with HQD (Additional file 1: Figure S1). As opposed to the DSS group, HQD significantly attenuated DSS-induced colon shortening (Fig. 1A, B). HQD treatment promoted weight recovery and reduced DAI scores (Fig. 1C, D). It repaired the damage in colon villous morphology and crypt caused by DSS while relieving colonic pathological states as evidenced by H&E staining (Fig. 1E). Quantitative PCR analysis demonstrated IL-6 and IL-1β, pro-inflammatory mediators, were downregulated in colonic tissues HQD-treated (Fig. 1F, G). Crucially, alleviating colitis symptoms exhibited HQD dose dependency, with high-dose HQD showing superior efficacy to low-dose (Fig. 1).

### HQD restored intestinal barrier function in DSS-induced mice

**Fig. 2:**
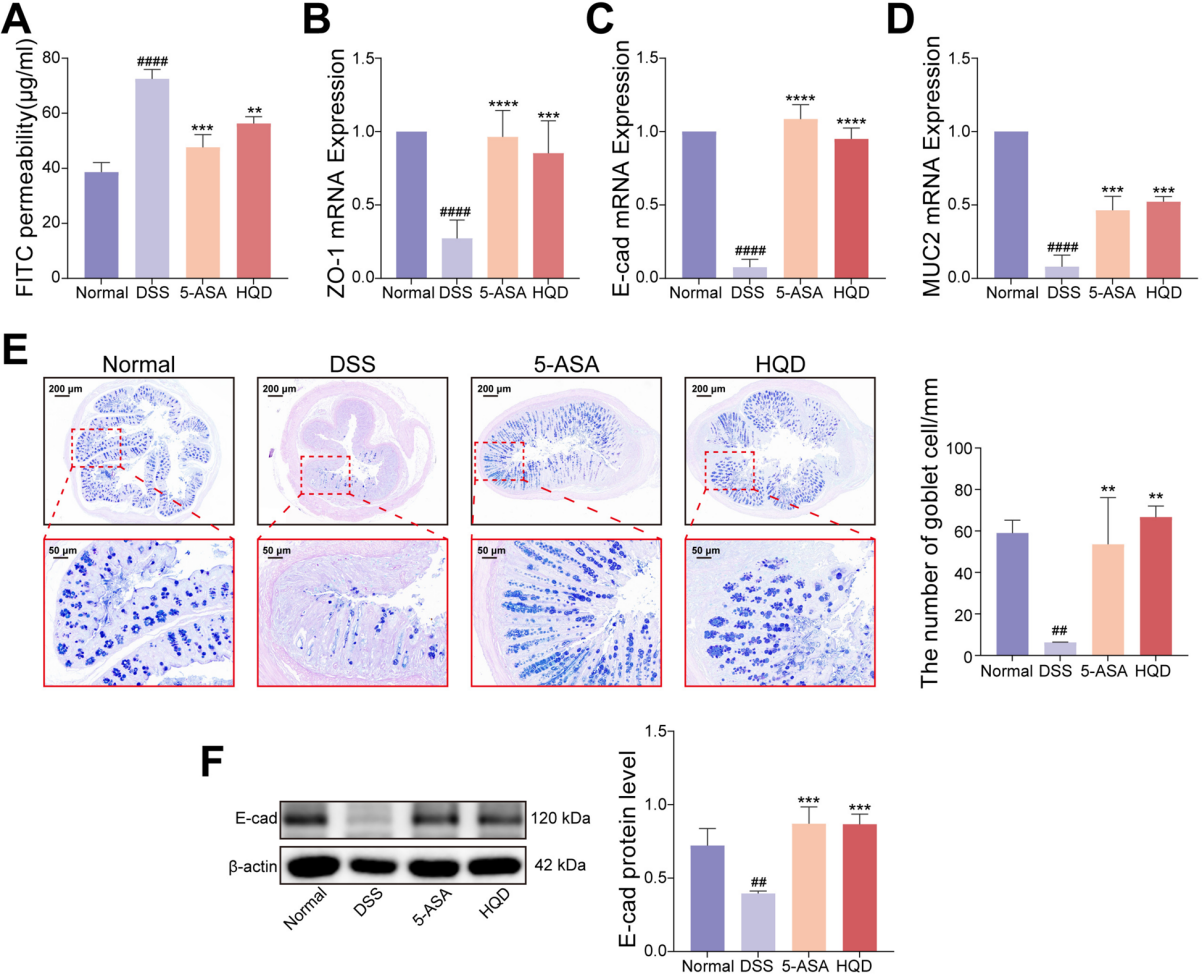
HQD restored intestinal barrier function in DSS-induced mice. **A** The intestinal permeability indicated by FITC-dextran concentration in mice serum. **B**–**D** The expression level of mRNA for ZO-1, E-cadherin, and MUC2. **E** The AB-PAS staining of colon. **F** The western blotting image and protein level of E-cadherin. Data were presented as mean ± SD (n = 3–5). ^##^*P* < 0.01, ^####^*P* < 0.0001, vs. Normal group. **P < 0.01, ***P < 0.001, ****P < 0.0001, vs. DSS group

Due to the superior efficacy of high-dose HQD compared to low-dose HQD, high-dose HQD (9.1 g/kg) was selected for further experiments. Intestinal barrier dysfunction represents a key feature of UC. To evaluate the restorative effects of HQD on barrier function, multiple indicators were assessed: HQD treatment reduced serum FITC-dextran concentrations in mice, indicating decreased intestinal permeability (Fig. 2A). Concurrently, HQD elevated gene expression levels of ZO-1 and E-cadherin (Fig. 2B, C), increased E-cadherin protein expression (Fig. 2F), enhanced MUC2 gene expression (Fig. 2D), and restored DSS-induced goblet cell depletion as shown by AB-PAS staining (Fig. 2E). These findings demonstrate that HQD promotes repair of the colonic mechanical and mucus barriers. Collectively, HQD protects the intestinal barrier of the colon in colitis.

### HQD treatment counteracts gut microbial dysbiosis

**Fig. 3:**
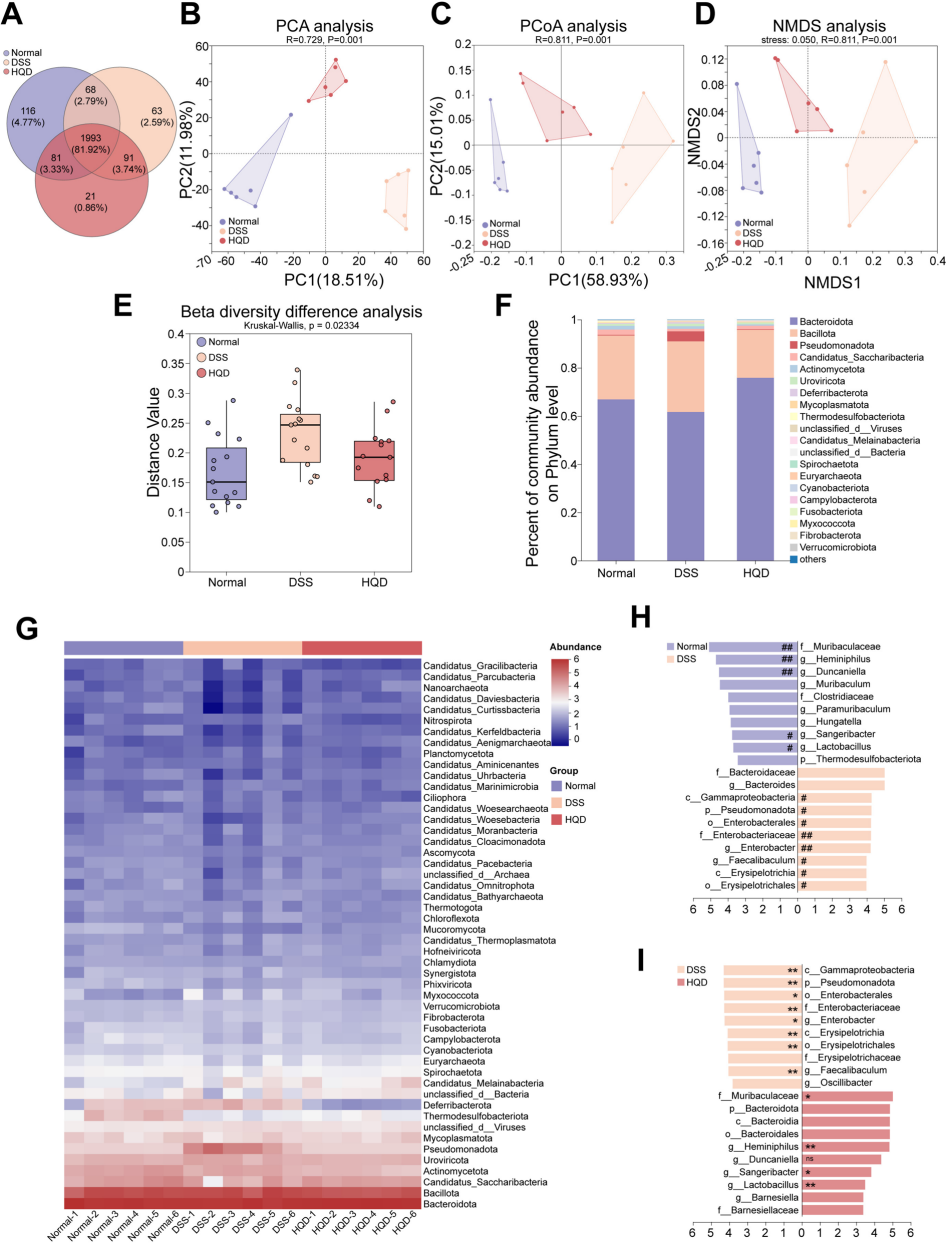
HQD treatment counteracts gut microbial dysbiosis. **A** Venn diagram showed shared genera comparison among the three groups. **B** PCA analysis. **C** PCoA analysis. **D** NMDS analysis. **E** β-diversity difference analysis. **F**, **G** Compositions and heatmap at the phylum level. **H**, **I** The top 10 keystone taxa (LDA score ≥ 3) per group with significance annotations using differential analysis across the three groups at the genus level. Data were presented as mean ± SD (n = 6). ^#^*P* < 0.05, ^##^*P* < 0.01, vs. Normal group. ^ns^*P* > 0.05, *P < 0.05, **P < 0.01, vs. DSS group

Venn analysis identified 1993 genera shared across all three groups, and compared to the Normal group and DSS group, the Normal group and HQD group had a higher genus overlap (Fig. 3A). Principal component analysis (PCA) assessed overall microbial community divergence as principal coordinates analysis (PCoA) and non-metric multidimensional scaling (NMDS) evaluated structural differences [14, 15]. β-diversity analysis determined inter-group significance [16]. At the genus level, PCA, PCoA, NMDS, and β-diversity revealed marked variations in gut bacterial communities’ structure across the three groups (Fig. 3B–E), indicating HQD increased bacterial abundance without altering microbiota specificity. Phylum-level histogram and heatmap demonstrated that HQD restored *Bacteroidota* abundance and reduced *Bacillota* levels versus the DSS group (Fig. 3F, G). Linear discriminant analysis (LDA) spanning phylum to genus level was performed between Normal vs. DSS groups and DSS vs. HQD groups, identifying the top 10 keystone taxa (LDA score ≥ 3) per group visualized in bar plots (Fig. 3H, I). Keystone taxa networks were constructed using Cytoscape (Additional file 1: Figure S2). Differential analysis among the three groups was performed with significance annotations (Fig. 3H, I). The above analyses identified *Muribaculaceae* (family level), *Heminiphilus* (genus level), *Sangeribacter* (genus level), and *Lactobacillus *(genus level) as shared keystone taxa in Normal and HQD groups, whereas DSS significantly reduced their abundance compared to the other groups. These results suggest HQD modulates gut microbiota homeostasis by restoring keystone taxa abundance.

### Effects of HQD on tryptophan metabolism and correlation with gut microbiota

**Fig. 4:**
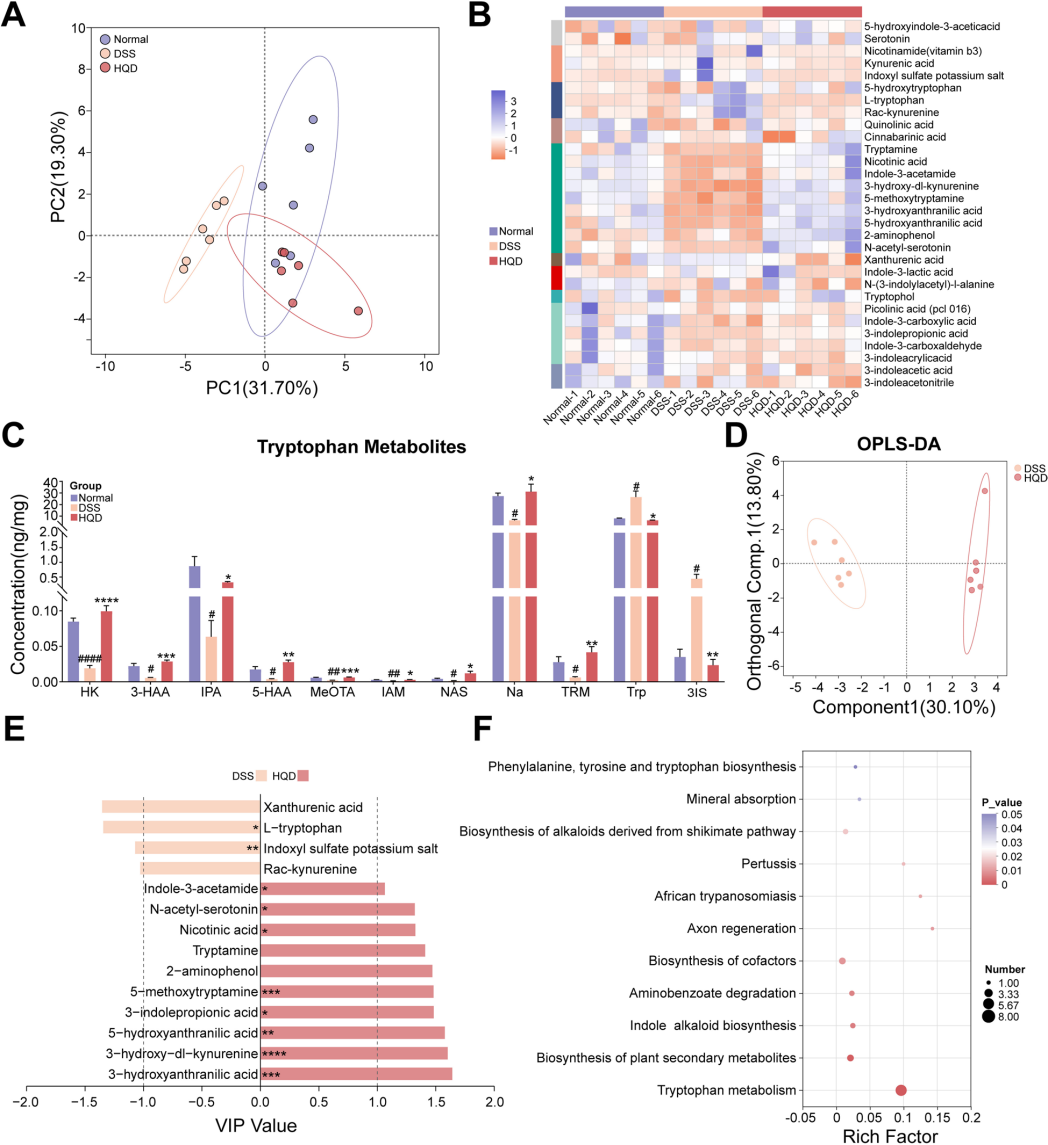
The effects of HQD on tryptophan metabolism. **A** PCA analysis. **B** The heatmap visualized the tryptophan metabolite profiles across all samples. **C** The differential analysis reuslt. Data were presented as mean ± SEM (n = 6). **D**, **E** The OPLS-DA analysis and VIP values. **F** The Enrichment analysis against the KEGG database was performed on differentially abundant metabolites between the DSS and HQD groups. ^#^*P* < 0.05, ^##^*P* < 0.01, ^####^*P* < 0.0001, vs. Normal group. *P < 0.05, **P < 0.01, ***P < 0.001, ****P < 0.0001, vs. DSS group

Dysbiosis of the gut microbiota often accompanies alterations in microbial metabolites. To elucidate whether and how HQD modulates tryptophan metabolism in colitic mice, we performed targeted metabolomic profiling of tryptophan and its derivatives in fecal samples. PCA revealed distinct clustering of tryptophan metabolic profiles among the three groups. Notably, the HQD group exhibited a composition more similar to that of Normal mice than that of DSS-induced mice (Fig. 4A). Furthermore, a clustered heatmap visualized the tryptophan metabolite profiles across all samples (Fig. 4B). Subsequent differential analysis identified HQD-regulated metabolites using the criteria of adjusted *P*-value (*P*.adj) < 0.05 and absolute log_2_fold-change (|log_2_FC|) ≥ 1 for the Normal vs. DSS and DSS vs. HQD comparisons (Fig. 4C). The separation of tryptophan metabolic profiles between the DSS and HQD groups was confirmed by Orthogonal partial least squares-discriminant analysis (OPLS-DA) (Fig. 4D). Metabolites with a variable importance in projection (VIP) score ≥ 1 were considered discriminant and visualized in a VIP score bar plot (Fig. 4E). Collectively, differential analysis and OPLS-DA demonstrated that HQD treatment enhanced L-tryptophan (Trp) metabolism compared to the DSS group. This enhancement manifested through the kynurenine, serotonin, and indole pathways, leading to increased levels of specific metabolites, including 3-hydroxyanthranilic acid (3-HAA), 5-methoxytryptamine (MeOTA), 5-hydroxyanthranilic acid (5-HAA), 3-hydroxy-DL-kynurenine (HK), Tryptamine (TRM), nicotinic acid (Na), indole-3-acetamide (IAM), and 3-indolepropionic acid (IPA). Among these, IPA, TRM, and IAM met the aforementioned differential criteria (P.adj < 0.05, |log_2_FC|≥ 1, VIP ≥ 1), as indole derivatives. Notably, indoxyl sulfate potassium salt (3IS) also met the above criterion but exhibited the inverse trend: it accumulated in the DSS group and was restored to lower levels by HQD. This directionality aligns with its established pro-inflammatory and gut-damaging properties [17]. Enrichment analysis against the KEGG database was applied to metabolites differing between DSS and HQD groups, which were calculated using the rich factor to gain functional insights. This analysis revealed significant enrichment in pathways including tryptophan metabolism, biosynthesis of plant secondary metabolites, indole alkaloid biosynthesis, and biosynthesis of alkaloids derived from shikimate pathway (Fig. 4F); thus, HQD treatment effectively modulated tryptophan metabolism in DSS-induced colitis mice, promoting the synthesis of alkaloids like indole derivatives.

**Fig. 5:**
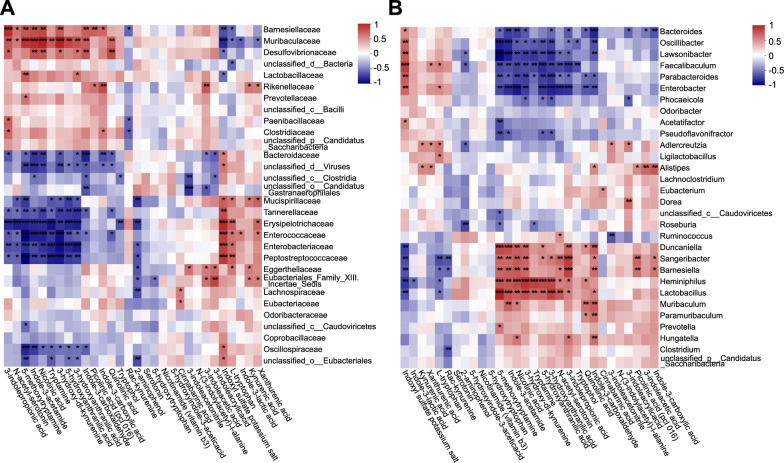
The spearman correlation analysis between gut microbiotas and tryptophan metabolites. **A** The heatmap of spearman correlation analysis at the family levels. **B** The heatmap of spearman correlation analysis at the genus levels. *P < 0.05, **P < 0.01, ***P < 0.001, n = 6

Collectively, HQD ameliorated DSS-induced gut microbiota dysbiosis and the disruption of tryptophan metabolism in mice. We performed Spearman correlation analysis to interrogate the association between alterations in microbial community structure and changes in indole-pathway metabolites—microbial products capable of activating AhR—across fecal samples from normal, DSS, and HQD groups. This analysis examined associations between gut microbial abundance (at the family and genus levels) and the concentrations of tryptophan metabolites across all samples; results are presented as a heatmap. At the family level, *Muribaculaceae* abundance correlated negatively with Trp and 3IS but positively with IPA, TRM, and IAM (Fig. 5A). At the genus level, *Heminiphilus*, *Sangeribacter*, and *Lactobacillus* each showed negative correlations with Trp and 3IS but positive correlations with IPA, TRM, and IAM (Fig. 5B). *Muribaculaceae* exhibited the strongest correlation with IAM, while *Heminiphilus*, *Sangeribacter*, and *Lactobacillus* were most closely linked to TRM, IPA, and IAM, respectively (Additional file 1: Table S5). Importantly, all correlations above were statistically significant, except for the positive correlation between *Sangeribacter* and TRM (Fig. 5).

### Blockade of AhR signalling compromises the ameliorative effects of HQD in colitis mice

**Fig. 6:**
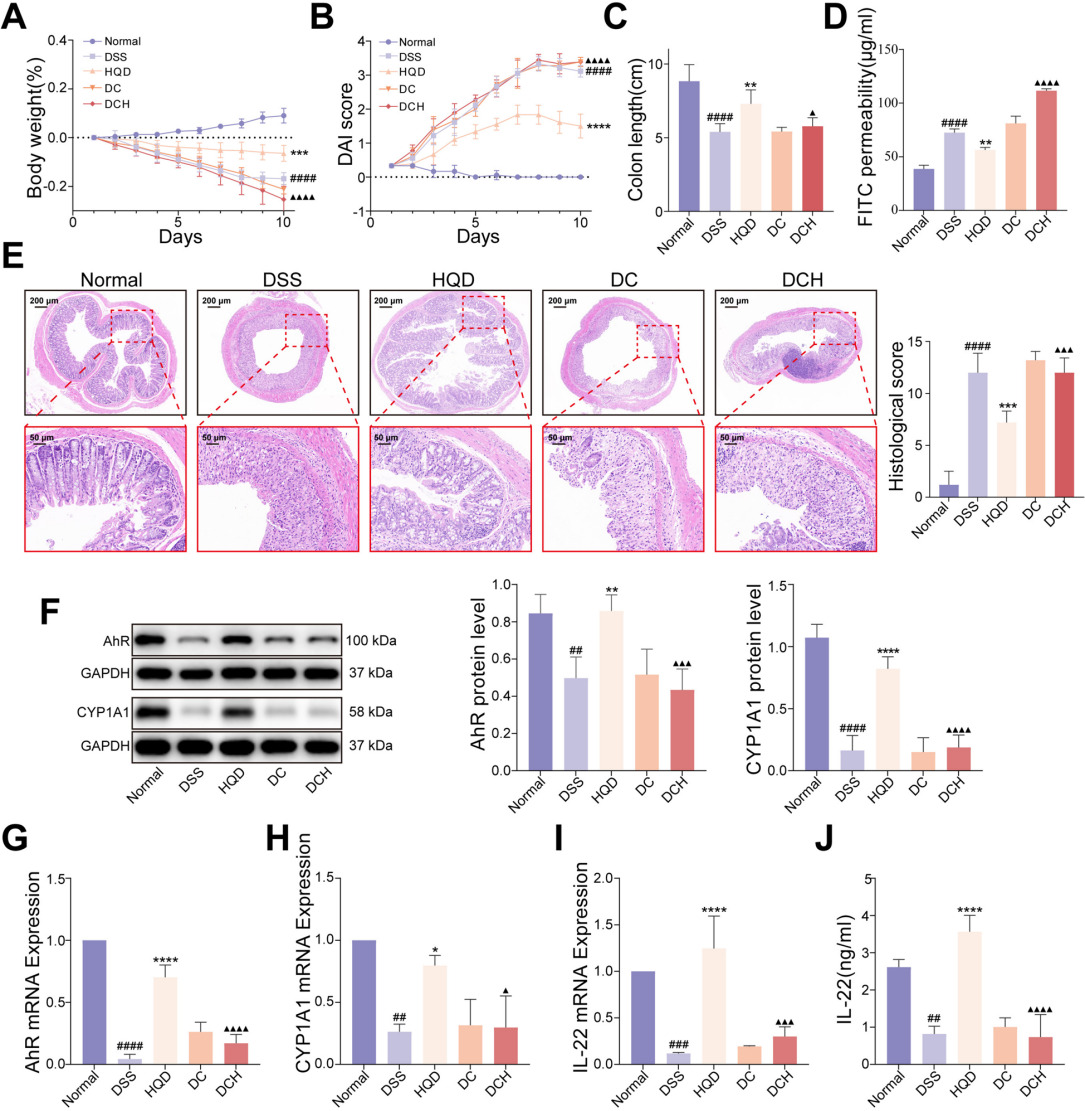
Blockade of AhR signalling compromises the ameliorative effects of HQD in colitis mice. **A** Body weight changes. **B** The DAI score. **C** The colon length of mice. **D** The intestinal permeability. **E** H&E staining. **F** AhR and CYP1A1 protein expression level. **G-I** AhR, CYP1A1, and IL-22 mRNA expression. **J** The content of IL-22 was evaluated using ELISA. Data were presented as mean ± SD (n = 3–6). ^##^*P* < 0.01, ^###^*P* < 0.001, ^####^*P* < 0.0001, vs. Normal group. *P < 0.05, **P < 0.01, ***P < 0.001, ****P < 0.0001, vs. DSS group. ^▲^*P* < 0.05, ^▲▲▲^*P* < 0.001, ^▲▲▲▲^*P* < 0.0001, vs. HQD group

To determine whether AhR signalling is indispensable for the therapeutic effects of HQD in DSS-colitis, we pharmacologically blocked the pathway by intraperitoneal administration of the selective antagonist CH223191. Consistent with our previous findings, HQD-treated mice were protected against DSS-induced body-weight loss, elevated disease activity index, shortened colon length, increased intestinal permeability, and histological damage. However, administration of CH223191 abolished these protective effects (Fig. 6A–E). Mechanistically, inhibitor CH223191 markedly down-regulated the mRNA and protein levels of AhR and its downstream targets CYP1A1 and IL22 in colonic tissue compared with HQD treatment alone, underscoring the critical role of AhR activation in HQD-mediated alleviation of experimental colitis (Fig. 6F–J).

### HQD regulates ISC differentiation by activating the AhR pathway in colitis

**Fig. 7:**
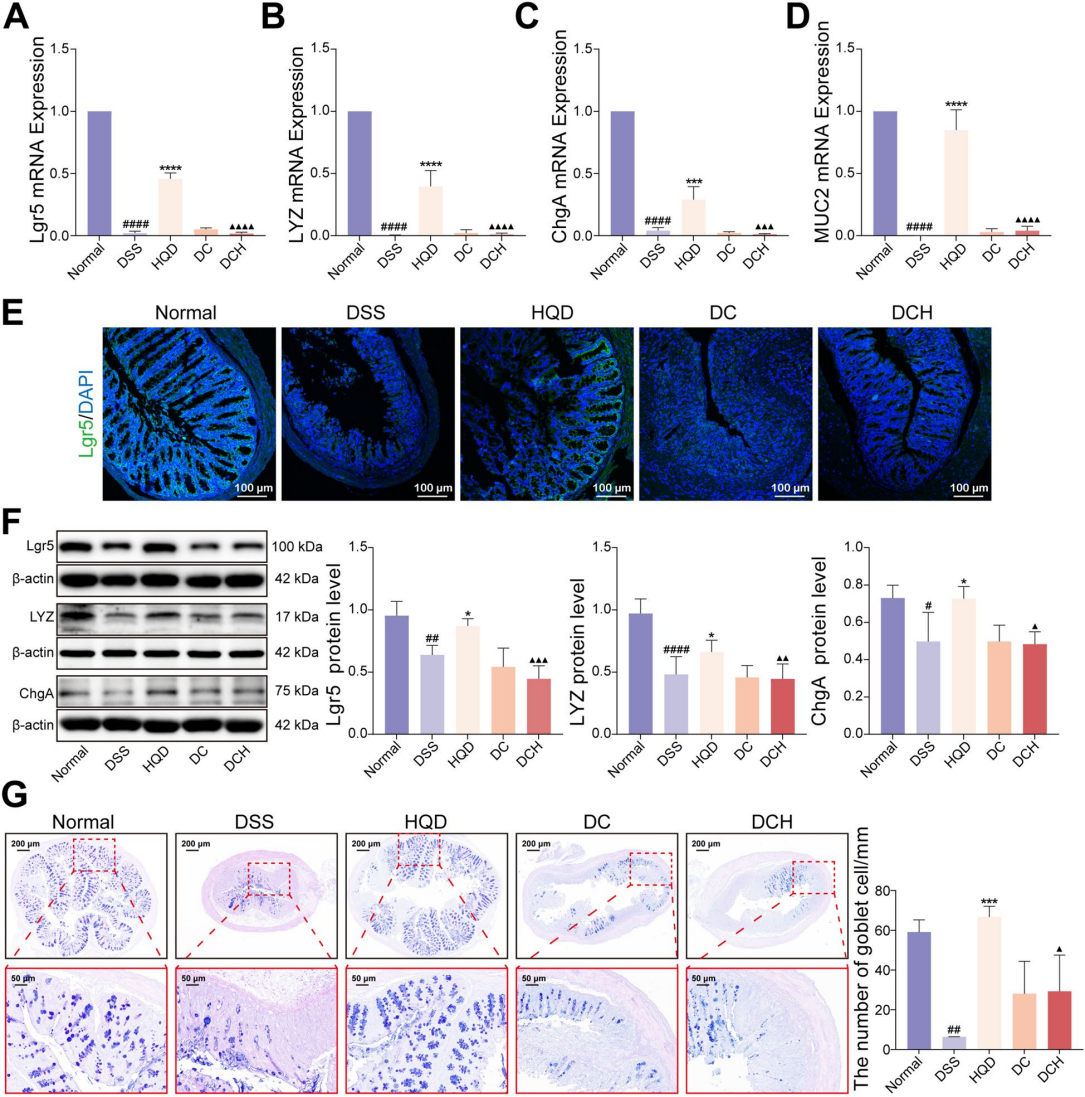
HQD regulates ISC differentiation by activating the AhR pathway in colitis. **A-D** Lgr5, LYZ, ChgA, and MUC2 mRNA expression. **E** Immunofluorescence image of Lgr5 in colon (Lgr5 staining, green; DAPI staining, blue). **F** Lgr5, LYZ, and ChgA protein expression levels. **G** AB-PAS staining. Data were presented as mean ± SD (n = 3–9). ^#^*P* < 0.05, ^##^*P* < 0.01, ^####^*P* < 0.0001, vs. Normal group. *P < 0.05, ***P < 0.001, ****P < 0.0001, vs. DSS group. ^▲^*P* < 0.05, ^▲▲^*P* < 0.01, ^▲▲▲^*P* < 0.001, ^▲▲▲▲^*P* < 0.0001, vs. HQD group

As shown above, pharmacologic AhR inhibition nullified the beneficial actions of HQD against DSS-provoked colitis. Given that AhR activation orchestrates ISC renewal and lineage commitment—processes indispensable for mucosal repair—we next asked whether HQD confers protection through an AhR-dependent ISC differentiation. RT-qPCR, Western blot, and immunofluorescence analyses revealed that, relative to DSS controls, HQD markedly up-regulated the mRNA levels of Lgr5 (ISC marker) and of its differentiated progeny markers MUC2 (goblet cells), ChgA (enteroendocrine cells), and LYZ (Paneth cells) (Fig. 7A–D). Concordantly, Lgr5, ChgA, and LYZ proteins were significantly increased (Fig. 7E–F). AB-PAS staining further demonstrated that HQD restored goblet-cell density (Fig. 7G). All these effects were almost entirely reversed by co-administration of the AhR antagonist CH223191 (Fig. 7). Collectively, these data establish that HQD alleviates murine colitis by activating AhR signaling to drive ISC differentiation.

### Microbiota depletion abrogates HQD-triggered activation of AhR and differentiation of ISC

**Fig. 8:**
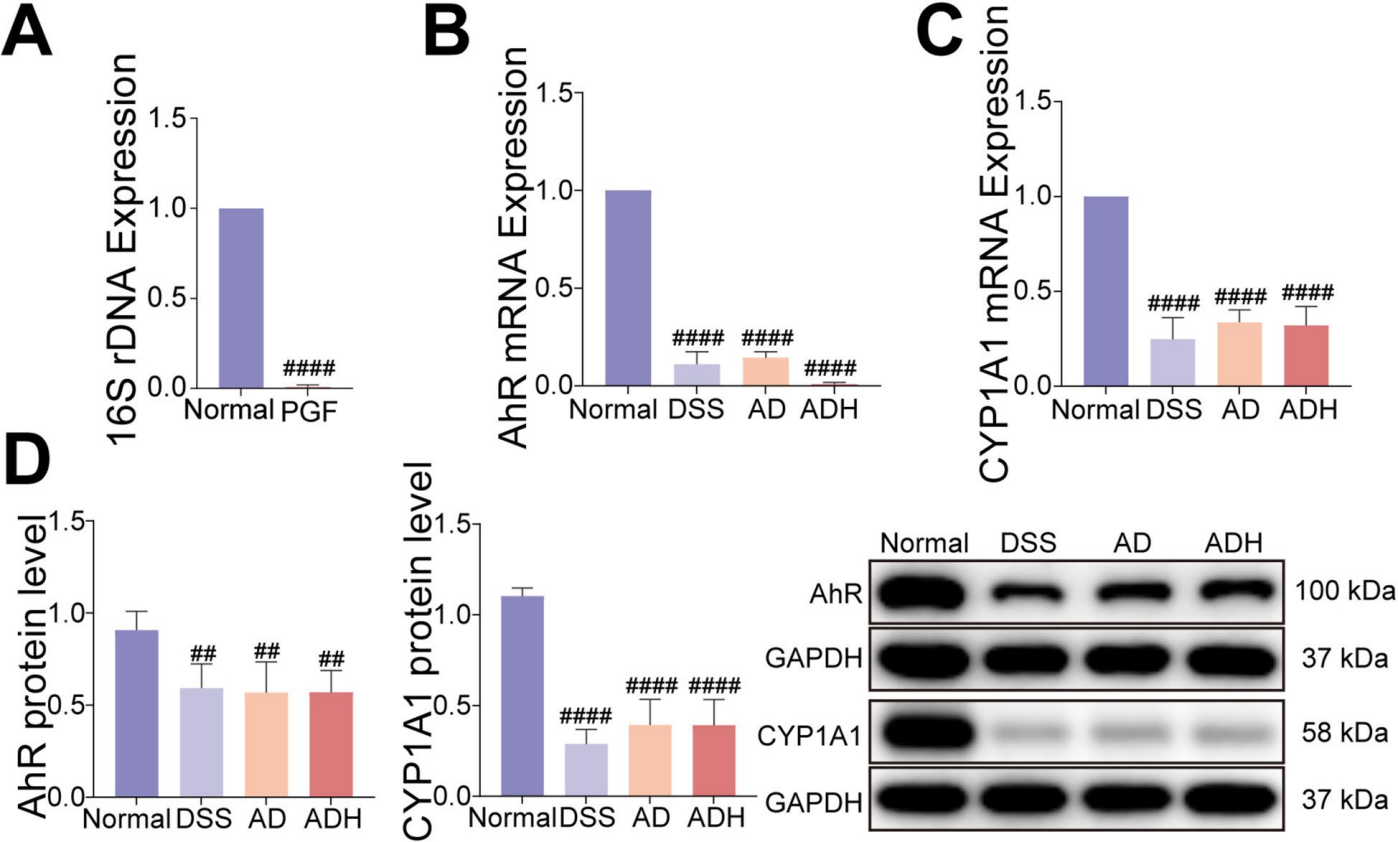
Microbiota depletion abrogates HQD-triggered activation of AhR. **A** Expression levels of 16S rDNA in fecal microorganisms of mice. **B**, **C** AhR and CYP1A1 mRNA expression. **D** AhR and CYP1A1 protein expression levels. Data were presented as mean ± SD (n = 6). ^##^*P* < 0.01, ^####^*P* < 0.0001, vs. Normal group

The preceding works demonstrate that HQD reshapes the gut microbiota to amplify tryptophan metabolism and that AhR antagonism abolishes HQD-induced ISC differentiation and colitis resolution. As the endogenous AhR ligands TAM, IPA, and IAM are microbially derived, we next asked whether an intact microbiota is obligatory for HQD to engage AhR and initiate ISC differentiation. Pseudo-germ-free (PGF) mice were generated with an Abx, and successful depletion was confirmed by > 99% reduction in faecal 16S rDNA copies (Fig. 8A).

**Fig. 9:**
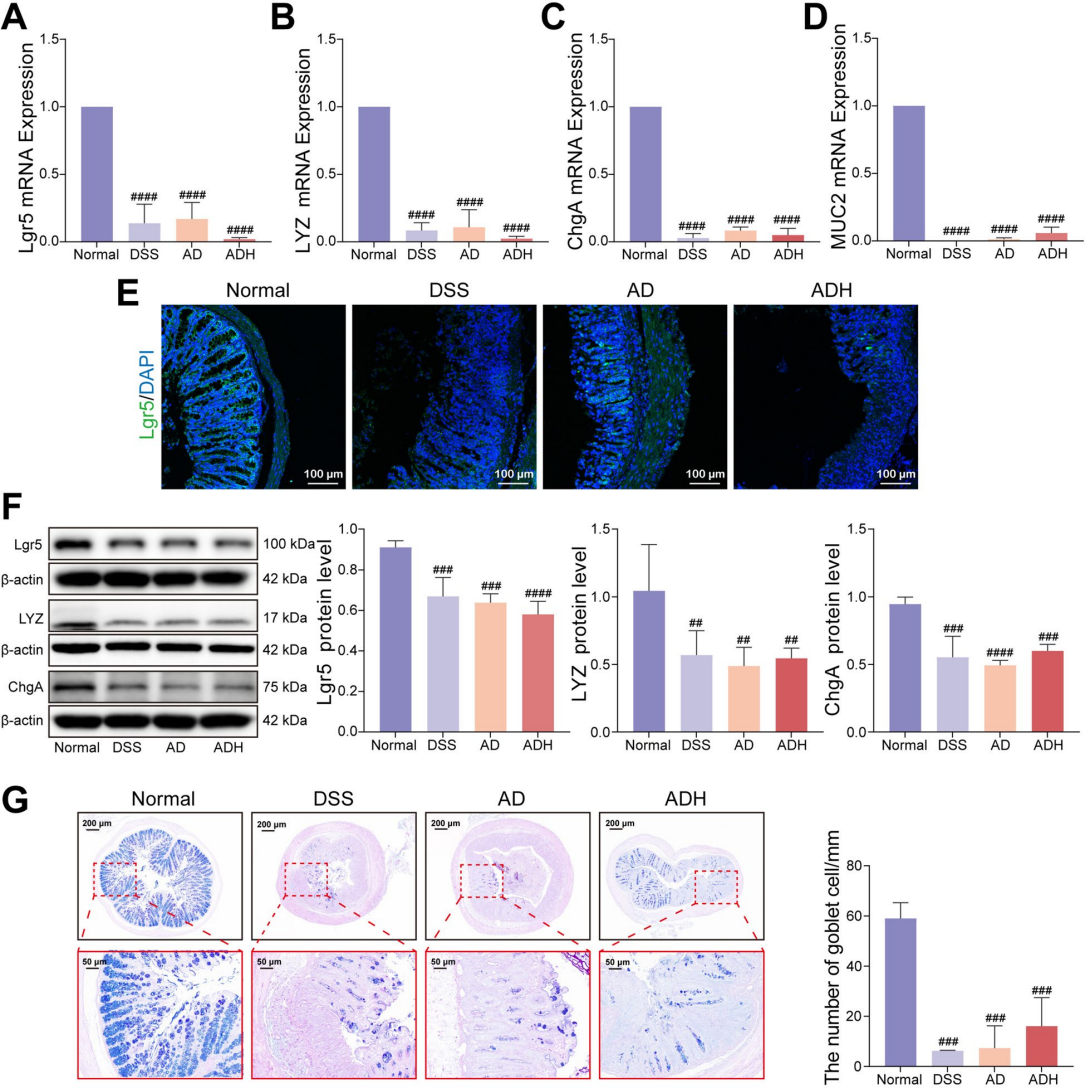
Microbiota depletion abrogates HQD-triggered differentiation of ISC. **A-D** Lgr5, LYZ, ChgA, and MUC2 mRNA expression. **E** Immunofluorescence image of Lgr5 in colon (Lgr5 staining, green; DAPI staining, blue,). **F** Lgr5, LYZ, and ChgA protein expression levels. **G** AB-PAS staining. Data were presented as mean ± SD (n = 6). ^##^*P* < 0.01, ^###^*P* < 0.001, ^####^*P* < 0.0001, vs. Normal group

Compared with the normal group, Abx-treated mice exhibited markedly attenuated HQD-induced up-regulation of colonic AhR and CYP1A1 at both mRNA and protein levels (Fig. 8B–D). Concomitantly, compared to the DSS group, Lgr5, ChgA, LYZ, and MUC2 transcripts, as well as Lgr5, ChgA, and LYZ proteins, failed to increase in AD and ADH groups (Fig. 9A–F). AB-PAS staining showed no restoration of goblet-cell numbers (Fig. 9G). Collectively, these data demonstrate that microbiota depletion prevents HQD from activating the AhR pathway, thereby abrogating its ability to promote ISC differentiation and mucosal repair in DSS colitis.

### Microbiota depletion compromises the ameliorative effects of HQD in colitis mice

**Fig. 10:**
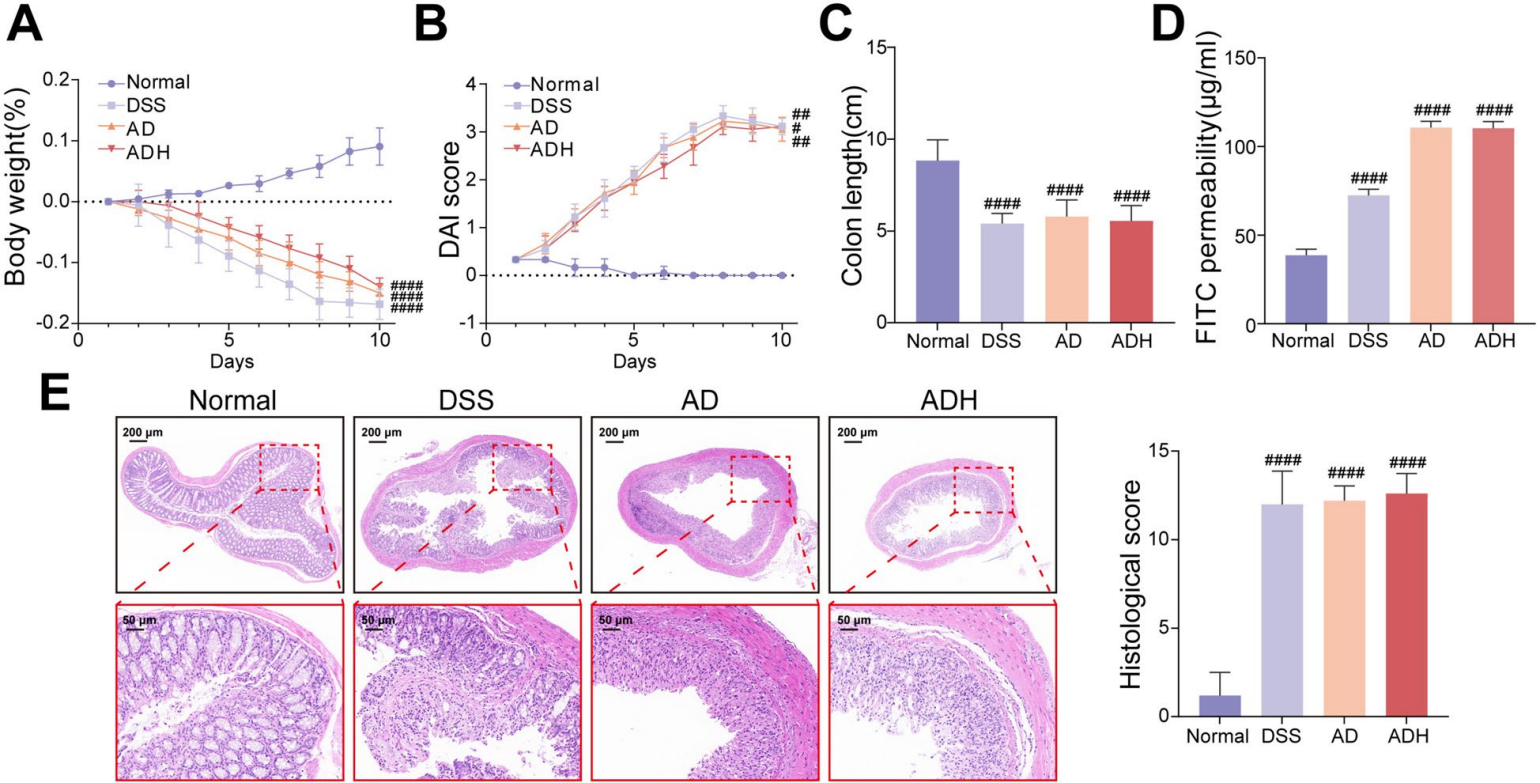
Microbiota depletion compromises the ameliorative effects of HQD in colitis mice. **A** Body weight changes. **B** The DAI score. **C** The colon length of mice. **D** The intestinal permeability. **E** H&E staining. Data were presented as mean ± SD (n = 3–6). ^#^*P* < 0.05, ^##^*P* < 0.01, ^####^*P* < 0.0001,vs Normal group

Consistent with the impaired HQD-triggered AhR-dependent ISC differentiation observed in AD and ADH groups, PGF mice failed to benefit from HQD. Body-weight loss, DAI score, colon length, intestinal permeability, and histopathological scores remained indistinguishable from the DSS group (Fig. 10), confirming that an intact microbiota is indispensable for HQD-mediated amelioration of colitis.

## Discussion

The incidence of UC, a persistent and non-specific inflammatory bowel disorder, is rising, posing an escalating global health challenge [18]. Accumulating evidence implicates dysbiotic gut microbiota and consequent epithelial-barrier failure as key pathogenic drivers [19]. HQD, a TCM, has repeatedly been shown to attenuate experimental and clinical colitis, and recent reports suggest that this benefit is mediated, at least in part, by microbial and amino-acid metabolomic reprogramming [13]. Here, we first validated a dose-dependent therapeutic efficacy of HQD in DSS-induced murine colitis and confirmed its ability to restore the compromised epithelial barrier. Nevertheless, the precise contribution of the microbiota and its metabolites, the causal relationships between specific taxa and bioactive metabolites, and the downstream molecular cascades elicited by microbiota-driven metabolites during HQD therapy remain ill-defined. The present study was therefore designed to dissect these unresolved questions.

The onset and development of ulcerative colitis are closely connected to gut microbiota. One of the consistent dysbiotic signatures in UC is an imbalanced *Bacillota/Bacteroidetes* (F/B) ratio, and both phyla have emerged as critical regulators of disease severity [20, 21]. Metagenomic profiling revealed that HQD administration shifted overall community structure away from the DSS-distorted state and restored a balanced F/B ratio; notably, the expansion of *Bacteroidetes* correlated strongly with clinical improvement [22]. Differential abundance and Linear discriminant analysis Effect Size further identified *Muribaculaceae*, *Heminiphilus*, *Sangeribacter*, and *Lactobacillus* as the core taxa shared between healthy and HQD-treated mice but markedly depleted in the DSS group. Independent studies have shown that enrichment of *Muribaculaceae* and *Lactobacillus* mitigates DSS-induced colitis [23, 24]. *Heminiphilus* and *Sangeribacter*, both nested within *Muribaculaceae *[25, 26], have likewise been characterized as health-associated symbionts: *Heminiphilus* may promote intestinal barrier repair [27], whereas *Sangeribacter* colonization does not exacerbate colonic inflammation [25]. These data indicate that HQD effectively reverses DSS-elicited dysbiosis by selectively restoring beneficial taxa—particularly *Muribaculaceae* and *Lactobacillus*—thereby re-establishing microbial homeostasis and supporting mucosal healing.

Gut microbial communities modulate host defense primarily through metabolite production rather than direct interactions with immune cells [28], like orchestrating Trp catabolism in the colon through decarboxylation, oxidation, and reduction, yielding indoles and their derivatives that exert anti-inflammatory effects, restore barrier integrity, and attenuate colitis [29, 30]. Moreover, indole metabolites are reduced in the mice with colitis induced by DSS [31]. In keeping with this, we found that DSS mice exhibited elevated fecal Trp and reduced indole derivatives, whereas HQD reversed these changes. Metabolomic profiling confirmed that HQD selectively enriched microbiota-derived indole alkaloids—IPA, TRM, and IAM—and pathway enrichment underscored indole alkaloid biosynthesis as the dominant Trp route activated by HQD. Spearman analysis revealed tight taxon–metabolite links. At the family level, *Muribaculaceae* abundance inversely correlated with Trp and positively correlated with IPA, TRM, and IAM. *Heminiphilus*, *Sangeribacter*, and *Lactobacillus* displayed identical trends at the genus level. And *Muribaculaceae, Heminiphilus*, *Sangeribacter*, and *Lactobacillus* were most closely linked to IAM, TRM, IPA, and IAM, respectively. Consistent with our in vivo findings, indole metabolites of tryptophan positively correlate with both *Lactobacillus* and *Muribaculaceae *[32]. Growth of *Lactobacillus* not only boosts IAM and indole-3-lactate generation but also potentiates AhR signalling and attenuates colitis [33, 34]. *Heminiphilus*, phylogenetically allied to *Muribaculum*, likely strengthens the genes of Trp metabolism [26, 35], while *Sangeribacter* encodes tryptophanase (EC 4.1.99.1), enabling direct hydrolysis of tryptophan to indole [25]. Thus, HQD restores Trp-to-indole flux by enriching taxa with validated or predicted tryptophanase activity, activating AhR-driven epithelial repair. One caveat is that *Muribaculaceae*, containing *Heminiphilus* and *Sangeribacter*, is largely murine-restricted; extrapolating this taxon-specific mechanism to humans requires validation in humanized or clinical cohorts.

Although indole and its metabolites are generally considered protective, they can also be detrimental. 3IS is a paradigmatic gut–host co-metabolite: colonic bacteria enzymatically convert dietary tryptophan to indole, which reaches the liver via the portal vein and is sulfated by cytochrome P450 and sulfotransferases to 3IS before renal excretion [36]. Beyond its well-documented nephrotoxicity, 3IS inhibits mitophagy, amplifies oxidative stress, inflicts epithelial injury, and aggravates mucosal inflammation [17, 37]. We observed that the relative abundances of *Muribaculaceae*, *Heminiphilus*, *Sangeribacter*, and *Lactobacillus* inversely correlated with faecal 3IS levels, which were elevated in DSS-treated mice and significantly reduced by HQD. Moreover, antibiotic-mediated microbiota ablation blunted HQD’s protection. These data suggest that HQD might mitigate 3IS-associated intestinal damage by modulating the microbiota to suppress 3IS production. Nevertheless, because 3IS synthesis along the gut–liver–kidney axis and its pro-inflammatory mechanisms are complex [38], they could not be addressed in the present study.

Certain indoles and their derivatives are endogenous AhR ligands, including IPA, TRM, and IAM [39–41], which could be produced upon HQD-induced reprogramming of tryptophan metabolism. AhR is constitutively expressed in intestinal epithelial cell (IEC) and immune cells, and its expression is reduced in intestinal tissue from IBD patients [6, 29]; moreover, AhR deficiency disrupts intestinal immunity and exacerbates colitis [29]. Restoring microbial AhR-ligand biosynthesis ameliorates DSS colitis [42], presumably because indole derivatives stimulate AhR in immune cells predominantly Group 3 Innate Lymphoid Cells (ILC3) to release IL-22 or activate AhR in IEC, thereby directing differentiation and function of ISC [43, 44]. IL-22, a pleiotropic cytokine that orchestrates intestinal immunoregulation and barrier restitution, is predominantly released by lamina propria lymphocytes—including ILC3, Gamma Delta T cells (γδ T cells), and select CD4^+^ T-helper subsets. Engagement of AhR within ILC3 and γδ T cells has been shown to potentiate IL-22 production [43]. Once liberated, IL-22 binds to the IL-22 receptor heterodimer on IEC, triggering STAT3 phosphorylation. Subsequent nuclear translocation of phosphorylated STAT3 drives transcriptional programs that accelerate proliferation, lineage specification, and regenerative differentiation of intestinal stem cells, thereby reinstating epithelial integrity [45]. Lgr5⁺ ISC possess multilineage potential, giving rise to Paneth, goblet, enteroendocrine, and absorptive cells that replenish mucus and rebuild the epithelial barrier [46]. Having established that HQD engages the microbiota–indole axis to restore barrier integrity and attenuate DSS colitis, we next asked whether this axis is linked to AhR activation and ISC differentiation. Compared with DSS group, both normal and HQD-treated mice exhibited elevated colonic expression of AhR and its downstream targets CYP1A1 and IL-22, alongside increased Lgr5 and lineage-specific markers (LYZ, ChgA, MUC2) and a higher goblet-cell density. Pharmacologic AhR blockade with CH223191 reversed these molecular and histological improvements and concurrently abolished HQD’s therapeutic benefit, indicating that AhR-driven Lgr5⁺ ISC differentiation is integral to HQD efficacy. Finally, in Abx-generated pseudo-germ-free mice, HQD failed to up-regulate AhR signaling or to promote Lgr5⁺ ISC differentiation, and it no longer offered protection against DSS-induced colitis. These data demonstrate that HQD requires a microbiota-derived indole pool to activate AhR and elicit Lgr5⁺ ISC-mediated mucosal repair.

HQD could alleviate DSS-induced colitis through microbiota-derived indoles that activate AhR and, directly or via IL-22, drive ISC differentiation; however, this attractive mechanistic framework is not unique to HQD—probiotics or synthetic AhR agonists can exploit the same pathway. However, probiotics demand individualized dosing and pose infection risks in immunocompromised hosts [47]. Similarly, chronic use of classical AhR agonists such as FICZ or TCDD can disturb immune homeostasis and provoke serious adverse effects (e.g., atherosclerosis, hepatic fibrosis) [48]. By contrast, HQD is easily formulated, offers natural chemical diversity, and simultaneously targets microbiota composition, Trp-metabolite reprogramming, and ISC differentiation. It therefore represents a comparatively simple, safe, and effective adjunctive therapy for ulcerative colitis.

**Fig. 11:**
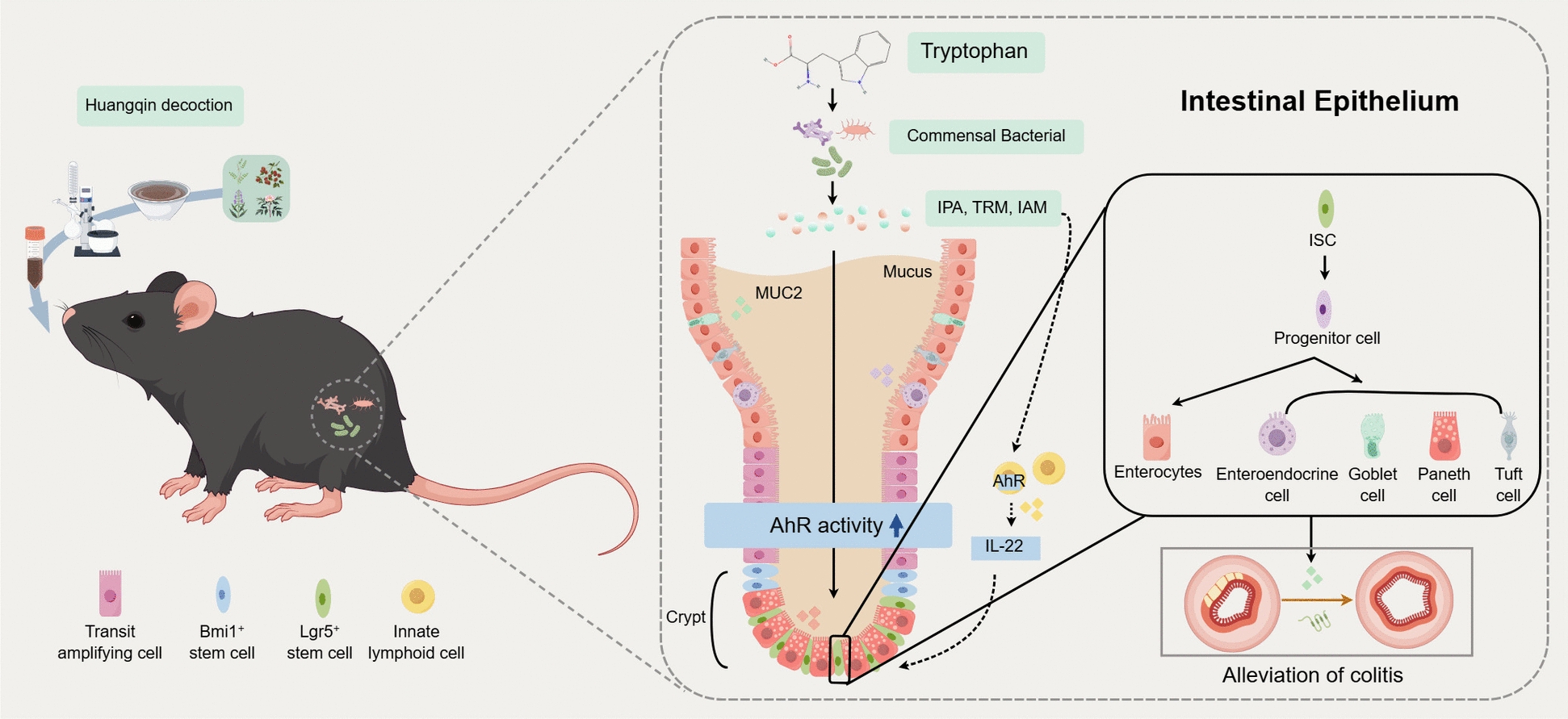
The experiment protocol designed used in this study was drawn by Figdraw

Although this study delineates core aspects of HQD’s action in colitis, several questions persist. Germ-free and AhR-knockout mice, together with gnotobiotic strain transplantation, are required to confirm the respective roles of the microbiota and AhR signalling in HQD-driven ISC differentiation. Our loss-of-function data implicate microbiota-derived tryptophan metabolites as essential drivers, yet they cannot rank IPA, TRM, or IAM by potency. Integrated AhR-reporter, organoid dose, and single-cell lineage-tracing assays should now enable the quantification of their individual contributions to secretory fate. The cellular source(s) of IL-22 within the inflamed mucosa remain to be identified by high-dimensional cytometry, and the mechanism by which HQD suppresses 3IS along the gut–liver–kidney axis merits targeted study. The experiments carried out in this research are depicted schematically in Fig. 11.

## Conclusion

In conclusion, indole and its derivatives exert dichotomous effects on intestinal homeostasis. HQD alleviates UC by repairing intestinal barrier integrity through AhR signaling-mediated ISC differentiation. This activation is likely mediated by gut microbiota-driven tryptophan metabolism and subsequent indole derivatives. Our study authenticates the traditional use of HQD and clarifies its pharmacological underpinnings by integrating UPLC-MS/MS quantification with multi-omics profiling, thereby advancing the modernization of this classical herbal formula. These data position HQD as a candidate microbiome-modulating therapy that merits prospective clinical validation.

## Supplementary Information

Additional file 1.

Additional file 2.

## Data Availability

The corresponding author can provide all data from this study upon a justified request.

## Abbreviations

AB-PAS: Alcian blue/periodic acid-Schiff
Abx: Antibiotic cocktail
AhR: Aryl hydrocarbon receptor
DAI: Disease activity index
DSS: Dextran sulfate sodium
ELISA: Enzyme-linked immunosorbent assay
FITC: Fluorescein isothiocyanate
H&E: Hematoxylin-eosin
HK: 3-Hydroxy-DL-kynurenine
HQD: Huangqin decoction
IAM: Indole-3-acetamide
IBD: Inflammatory bowel disease
IEC: Intestinal epithelial cell
IPA: 3-Indolepropionic acid
ISC: Intestinal stem cell
LDA: Linear discriminant analysis
MeOTA: 5-Methoxytryptamine
Na: Nicotinic acid
NMDS: Non-metric multidimensional scaling
OPLS-DA: Orthogonal partial least squares-discriminant analysis
PCA: Principal component analysis
PCoA: Principal coordinates analysis
PGF: Pseudo-germ-free
RT-qPCR: Reverse Transcription quantitative polymerase chain reaction
TCM: Traditional Chinese medicine
TRM: Tryptamine
Trp: L-tryptophan
UC: Ulcerative colitis
VIP: Variable importance in projection
3-HAA: 3-Hydroxyanthranilic acid
3IS: Indoxyl sulfate potassium salt
5-ASA: 5-Aminosalicylic acid
5-HAA: 5-Hydroxyanthranilic acid
γδ T: Gamma Delta T
