# Supplementary material for: Microbiota-driven tryptophan metabolism and AhR triggered intestinal stem cell differentiation: mechanisms of huangqin decoction in ulcerative colitis repair
Source: Chin Med. 2026 Jan 13;21:33. doi: 10.1186/s13020-025-01302-y (PMC12797797; doi:10.1186/s13020-025-01302-y)
Supplement: Supplementary file 1 — Additional file 1. [file 13020_2025_1302_MOESM1_ESM.pdf]

Microbiota-Driven Tryptophan Metabolism and AhR Triggered Intestinal Stem Cell Differentiation: Mechanisms of Huangqin Decoction in Ulcerative Colitis Repair.

Supplementary Material

Methods

Preparation and Composition of Huangqin decoction (HQD)

HQD was prepared by decocting a mixture of *Scutellaria baicalensis Georgi*, *Paeonia lactiflora Pall.*, *Glycyrrhiza uralensis Fisch.*, and *Ziziphus jujuba Mill.* (3:2:2:2 ratio) in pure water through two 30-minute extractions. The combined filtrates were concentrated to 1 g/mL and stored at 4 °C. Samples were thawed and centrifuged (13,800 × g, 4 °C, 15 min). Supernatant (300 μL) was mixed with 1000 μL extraction solvent (methanol:acetonitrile: water, 2:2:1, v/v/v) containing isotope-labeled internal standards. After vortexing (30 s) and ice-bath sonication (5 min), mixtures were incubated at -20 °C (1 h), then centrifuged again (13,800 × g, 4 °C, 15 min). The supernatant was filtered (0.22 μm) into vials.

Chromatography used a Vanquish UPLC system with a Kinetex C18 column (2.1 × 100 mm, 2.6 μm). Mobile phase: (A) 0.01% acetic acid/water, (B) isopropanol: acetonitrile (1:1, v/v); injection volume 2 μL at 4°C. Orbitrap Exploris 120 MS operated in DDA mode (Xcalibur v4.4): sheath/aux gas 50/15 arb, capillary 320°C, Full resolution 60K, MS1/MS2 resolution 15k, collision energy SNCE 20/30/40, spray voltage ±3.8/-3.4 kV.

Spearman correlation analysis

The Spearman correlation analysis were analyzed on the online tool of Majorbio Cloud Platform (https://www.majorbio.com/tools)[1].

Results

Identification of HQD components

An optimized UHPLC-HRMS workflow was established for comprehensive HQD chemical profiling. After stringent filtering (MS2_score ⩾ 3.5) and botanical annotation (Herb_id matching *cutellaria baicalensis Georgi*, *Paeonia lactiflora Pall.*, *Glycyrrhiza uralensis Fisch.*, and *Ziziphus jujuba Mill*), 112 components were identified (Table S6). Flavonoids account for approximately 40 % of the annotated compounds (Figure S3). Representative total-ion-current (TIC) chromatograms highlighted ten major bioactive components of HQD, including paeoniflorin, albiflorin, kaempferol, liquiritigenin, baicalin, catechin, wogonoside, oroxin A, quercetin, and licoisoflavone A (Figure S4).

References

1. Han C, Shi C, Liu L, Han J, Yang Q, Wang Y, et al. Majorbio Cloud 2024: Update single-cell and multiomics workflows. iMeta. 2024;3:e217.51

Supplementary Figure legends

Figure S1 Timeline of the animal experiment.

Figure S2 The Keystone taxa networks.

Figure S3 The class pie-chart distribution of the 112 HQD components identified by UHPLC-HRMS.

Figure S4 The UHPLC chromatograms of HQD. (A) Positive ion mode. (B) Negative ion mode.

Supplementary Figures

Figure S1

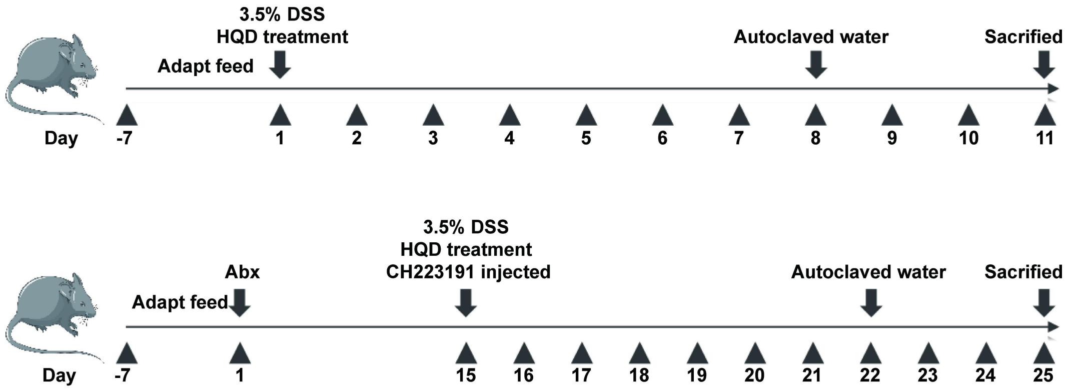

Figure S2

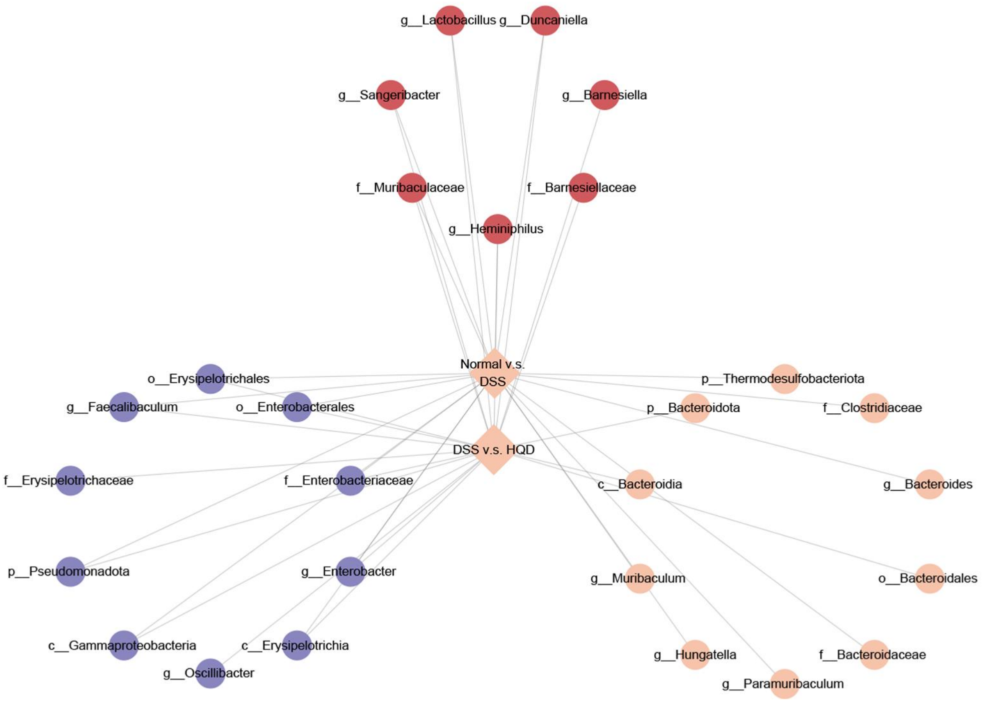
g__Lactobacillus
g__Duncaniella
g__Sangeribacter
g__Barnesiella
f__Muribaculaceae
f__Barnesiellaceae
g__Heminiphilus
Normal v.s. DSS
o__Erysipelotrichales
g__Faecalibaculum
o__Enterobacterales
p__Thermodesulfobacteriota
p__Bacteroidota
f__Clostridiaceae
DSS v.s. HQD
f__Erysipelotrichaceae
f__Enterobacteriaceae
c__Bacteroidia
g__Bacteroides
p__Pseudomonadota
g__Enterobacter
g__Muribaculum
o__Bacteroidales
c__Gammaproteobacteria
c__Erysipelotrichia
g__Oscillibacter
g__Hungatella
f__Bacteroidaceae
g__Paramuribaculum

Figure S3

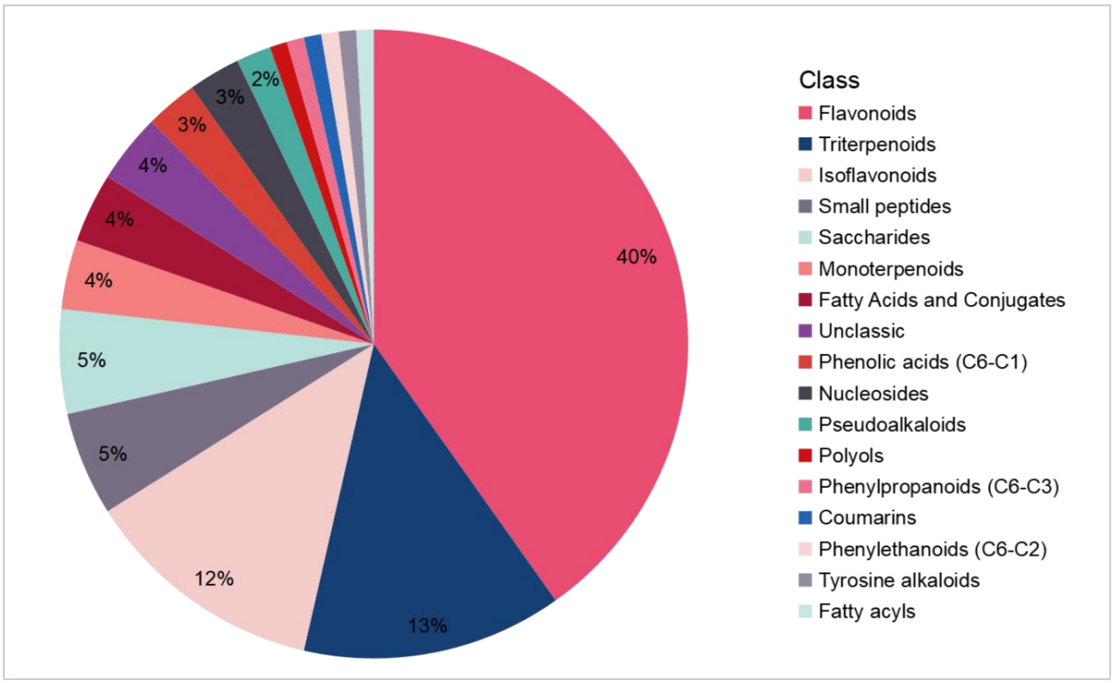
40%
13%
12%
5%
5%
4%
4%
4%
3%
3%
2%
Class
Flavonoids
Triterpenoids
Isoflavonoids
Small peptides
Saccharides
Monoterpenoids
Fatty Acids and Conjugates
Unclassic
Phenolic acids (C6-C1)
Nucleosides
Pseudoalkaloids
Polyols
Phenylpropanoids (C6-C3)
Coumarins
Phenylethanoids (C6-C2)
Tyrosine alkaloids
Fatty acyls

Figure S4

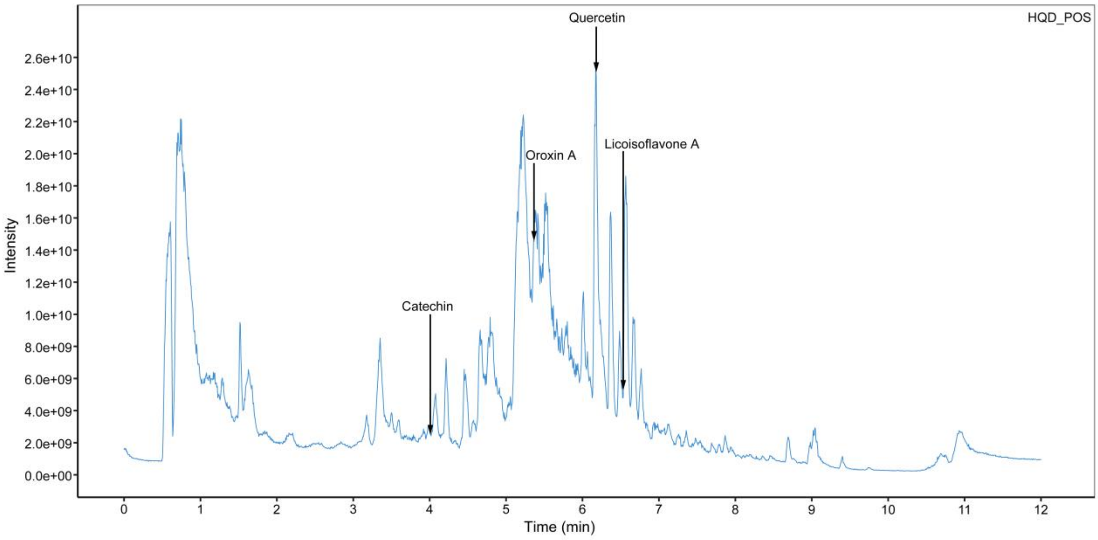

HQD_POS
Quercetin
Oroxin A
Licoisoflavone A
Catechin
Intensity
Time (min)

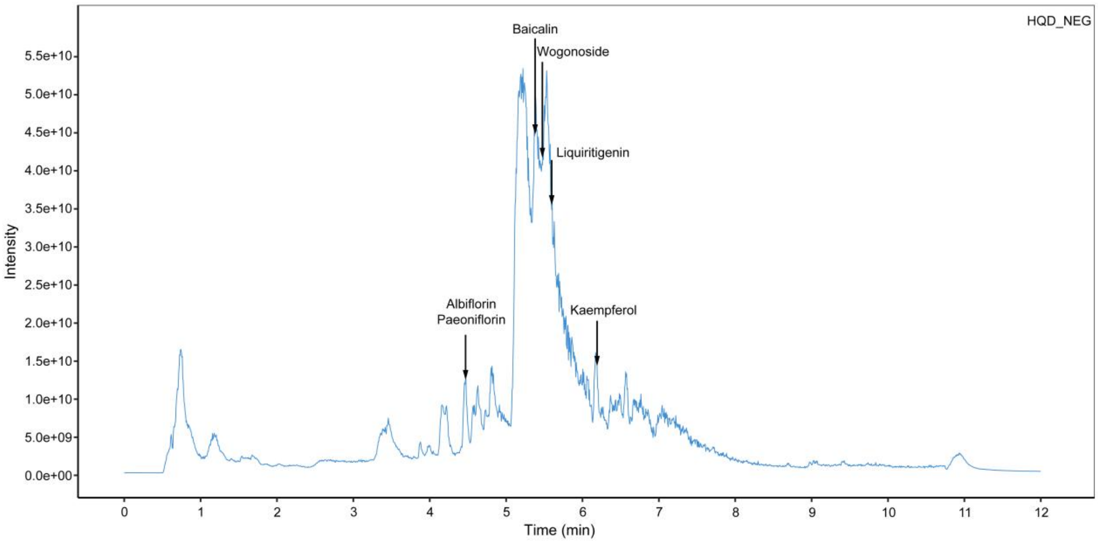

HQD_NEG
Baicalin
Wogonoside
Liquiritigenin
Albiflorin
Paeoniflorin
Kaempferol
Intensity
Time (min)

Supplementary Table

Table S1 The disease activity index scoring criteria

Score	Weight loss/%	Feces consistency	Hemafecia
0	0	Normal	Negative
1	1-5	Soft but well-formed	Slight bleeding
2	5-10	Soft and shapeless	Moderate bleeding
3	10-20	Very loose and moist	Gross bleeding
4	>20	Diarrhea	Blood clot around anus

Table S2 The histopathological scoring criteria

Grade	Severity	Description
0	Normal	Tissue is considered normal under study conditions that account for age, sex, and strain of the animal; any deviations under these defined conditions are deemed abnormal.
1	Very minimal	Changes are barely beyond the normal range.
2	Mild	Lesions are present but not pronounced.
3	Moderate	Obvious lesions that are expected to progress to a more severe state.
4	Severe	Marked, extensive lesions occupying most or all of the tissue/organ.

Table S3 The Chromatographic gradient

Time(min)	flow velocity(mL/min)	A%	B%
0.0	0.35	99	1
2.5	0.35	89	11
5.5	0.35	89	11
6.5	0.35	72	28
7.5	0.35	72	28
12.5	0.35	50	50
13.5	0.35	5	95
15.5	0.35	5	95
15.6	0.35	99	1
18.0	0.35	99	1

Table S4 The Primer sequences used in RT-qPCR

Species	Gene	Forward primer(5'-3')	Reverse primer(5'-3')
Mouse	ZO-1	GCTTTAGCGAACAGAAGGAGC	TTCATTTTTCCGAGACTTCACCA
Mouse	IL-6	TAGTCCTTCCTACCCCAATTTCC	TTGGTCCTTAGCCACTCCTTC
Mouse	IL-1β	GAAATGCCACCTTTTGACAGTG	TGGATGCTCTCATCAGGACAG
Mouse	E-cadherin	CAGGTCTCCTCATGGCTTTGC	CTTCCGAAAAGAAGGCTGTCC
Mouse	IL-22	ATGAGTTTTTCCCTTATGGGGAC	GCTGGAAGTTGGACACCTCAA
Mouse	CYP1A1	GGGTTTGACACAGTCACAACT	GGGACGAAGGATGAATGCCG
Mouse	AhR	AGCCGGTGCAGAAAACAGTAA	AGGCGGTCTAACTCTGTGTTC
Mouse	Lgr5	CCTACTCGAAGACTTACCCAGT	GCATTGGGGTGAATGATAGCA
Mouse	LYZ	GGAATGGATGGCTACCGTGG	CATGCCACCCATGCTCGAAT
Mouse	ChgA	ATCCTCTCTATCCTGCGACAC	GGGCTCTGGTTCTCAAACACT
Mouse	MUC2	AACGATGCCTACACCAAGGTC	ACTGAACTGTATGCCTTCCTCA
Mouse	GAPDH	TGACCTCAACTACATGGTCTACA	CTTCCCATTCTCGGCCTTG
Mouse	Actb	ATGACCCAGATCATGTTTGA	TACGACCAGAGGCATACAG
Bateria	16s rDNA	AGAGTTTGATCATGGCTCAG	TGCTGCCTCCCGTAGGAGT

Table S5 The correlation coefficient (r) of Spearman correlation analysis

	Family	Genus		
	Muribaculaceae	*Heminiphilus*	*Sangeribacter*	*Lactobacillus*
L-tryptophan	-0.488132095	-0.488132095	-0.628482972	-0.496388029
Indole-3-acetamide	0.777089783	0.696594427	0.622291022	0.708978328
3-indolepropionic acid	0.624355005	0.562435501	0.795665635	0.537667699
Tryptamine	0.609907121	0.795665635	0.374613003	0.632610939
Indoxyl sulfate potassium salt	-0.731682147	-0.702786378	-0.626418989	-0.614035088

Table S6 Chromatographic and mass spectral data of compounds analyzed by UHPLC-HRMS

MS2_name	Formula	Class	MS2_score	mzmed	rtmed	ms2Adduct
Paeoniflorin	C23H28O11	Monoterpenoids	3.99	479.1559	268.1	[M-H]-
Valine	C5H11NO2	Small peptides	3.99	116.0717	46.7	[M-H]-
Myristic acid	C14H28O2	Fatty Acids and Conjugates	3.98	227.2019	537.5	[M-H]-
Glycyrrhizin	C42H62O16	Triterpenoids	3.98	821.3971	434.6	[M-H]-
Licorice-saponin H2	C42H62O16	Triterpenoids	3.98	821.3971	434.6	[M-H]-
Diammonium Glycyrrhizinate	C42H62O16	Triterpenoids	3.98	821.3971	434.6	[M-H]-
Choerospondin	C21H22O10	Flavonoids	3.98	433.1144	307.4	[M-H]-
L-Proline	C5H9NO2	Small peptides	3.98	114.0561	46	[M-H]-
Ursolic acid	C30H48O3	Triterpenoids	3.97	455.3536	530.1	[M-H]-
D-Proline	C5H9NO2	Small peptides	3.97	114.0561	46	[M-H]-
Sucrose	C12H22O11	Saccharides	3.97	341.1087	44.9	[M-H]-
Corosolic acid	C30H48O4	Triterpenoids	3.97	471.3486	494.8	[M-H]-
(1S,2R,4aS,6aS,6bR,10S,11R,12aR,14bS)-10,11-dihydroxy-1,2,6a,6b,9,9,12a-heptamethyl-2,3,4,5,6,6a,7,8,8a,10,11,12,13,14b-tetradecahydro-1H-picene-4a-carboxylic acid	C30H48O4	Triterpenoids	3.97	471.3486	494.8	[M-H]-
Pelargonic acid	C9H18O2	Fatty Acids and Conjugates	3.97	157.1235	449.4	[M-H]-
Leucine	C6H13NO2	Small peptides	3.97	130.0875	64.4	[M-H]-
(1S,2R,4aS,6aS,6bR,10R,11R,12aR,14bS)-10,11-dihydroxy-1,2,6a,6b,9,9,12a-heptamethyl-2,3,4,5,6,6a,7,8,8a,10,11,12,13,14b-tetradecahydro-1H-picene-4a-carboxylic acid	C30H48O4	Triterpenoids	3.96	471.3486	494.8	[M-H]-
Benzoic acid	C7H6O2	Phenolic acids (C6-C1)	3.96	121.0295	318.7	[M-H]-
Vicenin-1	C26H28O14	Flavonoids	3.96	563.141	269.2	[M-H]-
Sebacic acid	C10H18O4	Fatty Acids and Conjugates	3.96	201.1133	352.5	[M-H]-
Galactose	C6H12O6	Saccharides	3.95	203.0521	41.5	[M+Na]+
18α-Glycyrrhetinic acid	C30H46O4	Triterpenoids	3.95	469.3327	493.5	[M-H]-
Licoricone	C22H22O6	Isoflavonoids	3.95	381.1345	427.7	[M-H]-
Scutellarin	C21H18O12	Flavonoids	3.95	461.0727	298.4	[M-H]-

Rutin	C27H30O16	Flavonoids	3.95	609.1464	285.7	[M-H]-
7-hydroxy-2-[4-[(2S,3R,4S,5S,6R)-3,4,5-trihydroxy-6-(hydroxymethyl)tetrahydropyran-2-yl]oxyphenyl]chroman-4-one	C21H22O9	Flavonoids	3.95	417.119	284.5	[M-H]-
(2R,3R)-2-(2,6-dihydroxyphenyl)-3,5,7-trihydroxy-chroman-4-one	C15H12O7	Flavonoids	3.95	303.0514	262.9	[M-H]-
Asparagine	C4H8N2O3	Small peptides	3.95	131.0464	41.1	[M-H]-
5,7-dihydroxy-2-[4-[3,4,5-trihydroxy-6-(hydroxymethyl)tetrahydropyran-2-yl]oxyphenyl]chroman-4-one	C21H22O10	Flavonoids	3.94	435.1275	300.9	[M+H]+
Albiflorin	C23H28O11	Monoterpenoids	3.94	479.1559	268.1	[M-H]-
Allose	C6H12O6	Saccharides	3.94	179.0562	41.8	[M-H]-
Mannose	C6H12O6	Saccharides	3.94	179.0562	41.8	[M-H]-
Schaftoside	C26H28O14	Flavonoids	3.94	563.141	269.2	[M-H]-
(2S,4aS,6aS,6bR,10S,12aS,14bS)-10-hydroxy-2,4a,6a,6b,9,9,12a-heptamethyl-13-oxo-3,4,5,6,6a,7,8,8a,10,11,12,14b-dodecahydro-1H-picene-2-carboxylic acid	C30H46O4	Triterpenoids	3.94	469.3327	493.5	[M-H]-
Isoliquiritigenin	C15H12O4	Flavonoids	3.94	255.0665	375.5	[M-H]-
Kaempferol	C15H10O6	Flavonoids	3.94	285.0406	371.2	[M-H]-
Liquiritigenin	C15H12O4	Flavonoids	3.94	255.0663	335.8	[M-H]-
Baicalin	C21H18O11	Flavonoids	3.94	445.0775	322.8	[M-H]-
Phenol	C6H6O		3.94	93.0346	294.7	[M-H]-
Malic acid	C4H6O5	Fatty Acids and Conjugates	3.94	133.0144	66.9	[M-H]-
Semilicoisoflavone B	C20H16O6	Isoflavonoids	3.93	353.1007	406.9	[M+H]+
5,7,11,19-tetraoxapentacyclo[10.8.0.02,10.04,8.013,18]icosa-2,4(8),9,13(18),14,16-hexaen-16-ol	C16H12O5	Isoflavonoids	3.93	285.0747	377.6	[M+H]+
Cosmosiin	C21H20O10	Flavonoids	3.93	433.1113	306.3	[M+H]+
[(1S,2S,3R,5R,6S,8S)-6-hydroxy-8-methyl-3-[(2S,3R,4S,5S,6R)-3,4,5-trihydroxy-6-(hydroxymethyl)tetrahydropyran-2-yl]oxy-9,10-dioxatetracyclo[4.3.1.02,5.03,8]decan-2-yl]methyl benzoate	C23H28O11	Monoterpenoids	3.93	479.1559	268.1	[M-H]-
(E)-3-[5-(1,1-dimethylallyl)-4-hydroxy-2-methoxy-phenyl]-1-(4-hydroxyphenyl)prop-2-en-1-one	C21H22O4	Flavonoids	3.93	337.1448	428.6	[M-H]-
2-[4-[3-[3,4-dihydroxy-4-(hydroxymethyl)tetrahydrofuran-2-yl]oxy-4,5-dihydroxy-6-(hydroxymethyl)tetrahydropyran-2-yl]oxyphenyl]-7-hydroxy-chroman-4-one	C26H30O13	Flavonoids	3.93	549.1615	275.9	[M-H]-

Medicarpin	C16H14O4	Isoflavonoids	3.92	271.0954	385.4	[M+H]+
[(2R,3S,4S,5R,6S)-6-[[(1S,3R,5R,6S,8S)-2-(benzoyloxymethyl)-6-hydroxy-8-methyl-9,10-dioxatetra cyclo[4.3.1.02,5.03,8]decan-3-yl]oxy]-3,4,5-trihydroxy-tetrahydropyran-2-yl]methyl benzoate	C30H32O12	Monoterpenoids	3.92	585.1942	345	[M+H]+
Vicenin 2	C27H30O15	Flavonoids	3.92	595.1648	257	[M+H]+
Catechin	C15H14O6	Flavonoids	3.92	291.0857	240.6	[M+H]+
3-Epiursolic Acid	C30H48O3	Triterpenoids	3.92	455.3536	530.1	[M-H]-
Sorbose	C6H12O6	Saccharides	3.92	179.0562	41.8	[M-H]-
Pyrogallol	C6H6O3		3.92	125.0245	102.2	[M-H]-
Wogonoside	C22H20O11	Flavonoids	3.92	459.0932	328.8	[M-H]-
Pyrocatechol	C6H6O2		3.92	109.0296	219.9	[M-H]-
D-Pinitol	C7H14O6	Polyols	3.92	193.0719	45.2	[M-H]-
Isoformononetin	C16H12O4	Isoflavonoids	3.91	269.0798	373.8	[M+H]+
(-)-Catechin	C15H14O6	Flavonoids	3.91	291.0857	240.6	[M+H]+
Licoricidin	C26H32O5	Isoflavonoids	3.91	425.2311	453.6	[M+H]+
5-Hydroxy-7,8-dimethoxyflavone,Moslosooflavone	C17H14O5	Flavonoids	3.91	299.0904	415.2	[M+H]+
Ononin	C22H22O9	Isoflavonoids	3.91	431.1318	316.8	[M+H]+
Riboflavin	C17H20N4O6	Pseudoalkaloids	3.91	377.1448	249.7	[M+H]+
6,8-Diprenylgenistein	C25H26O5	Isoflavonoids	3.91	405.1711	486.6	[M-H]-
Vestitol	C16H16O4	Isoflavonoids	3.91	271.0977	375.5	[M-H]-
1-phenylbutane-1,3-dione	C10H10O2		3.9	163.0748	454.9	[M+H]+
7-hydroxy-2-(4-hydroxyphenyl)chroman-4-one	C15H12O4	Flavonoids	3.9	255.0663	335.8	[M-H]-
(2R)-5,7-dihydroxy-2-(4-hydroxyphenyl)-6-methoxy-chroman-4-one	C16H14O6	Flavonoids	3.9	301.0719	345.9	[M-H]-
2-(2,6-dihydroxyphenyl)-3,5,7-trihydroxy-chromone	C15H10O7	Flavonoids	3.9	301.0357	307.4	[M-H]-
4-Hydroxycinnamic acid	C9H8O3	Phenylpropanoids (C6-C3)	3.9	163.0401	293.6	[M-H]-
Methylgallate	C8H8O5	Phenolic acids (C6-C1)	3.9	183.0301	243.2	[M-H]-
5,7-dihydroxy-2-(4-hydroxyphenyl)-6,8-bis[3,4,5-trihydroxy-6-(hydroxymethyl)tetrahydropyran-2-yl]chromen-4-one	C27H30O15	Flavonoids	3.89	595.1648	257	[M+H]+

4-[7-hydroxy-5-methoxy-6-(3-methylbut-2-enyl)chroman-3-yl]benzene-1,3-diol	C21H24O5	Isoflavonoids	3.89	357.1685	405.9	[M+H]+
Oroxin A	C21H20O10	Flavonoids	3.89	433.111	322	[M+H]+
5,7-dihydroxy-2-(4-hydroxy-3-methoxy-phenyl)-3-[3,4,5-trihydroxy-6-[[(2R,3R,4R,5R,6S)-3,4,5-trihydroxy-6-methyl-tetrahydropyran-2-yl]oxymethyl]tetrahydropyran-2-yl]oxy-chromen-4-one	C28H32O16	Flavonoids	3.89	625.1746	299.2	[M+H]+
(4aS,6aS,6bR,9R,10S,12aR,14bS)-10-hydroxy-9-(hydroxymethyl)-2,2,6a,6b,9,12a-hexamethyl-1,3,4,5,6,6a,7,8,8a,10,11,12,13,14b-tetradecahydropicene-4a-carboxylic acid	C30H48O4	Triterpenoids	3.89	471.3486	494.8	[M-H]-
5,7-dihydroxy-2-(4-hydroxyphenyl)-8-[3,4,5-trihydroxy-6-(hydroxymethyl)tetrahydropyran-2-yl]-6-(3,4,5-trihydroxytetrahydropyran-2-yl)chromen-4-one	C26H28O14	Flavonoids	3.89	563.141	269.2	[M-H]-
2-(3,4-dihydroxyphenyl)-5,7-dihydroxy-6,8-dimethoxy-chromen-4-one	C17H14O8	Flavonoids	3.89	345.0614	318.7	[M-H]-
4-[(3R)-5,7-dimethoxy-6-(3-methylbut-2-enyl)chroman-3-yl]-2-(3-methylbut-2-enyl)benzene-1,3-diol	C27H34O5	Isoflavonoids	3.87	439.2468	485.5	[M+H]+
7-hydroxy-2-[4-hydroxy-3-(3-methylbut-2-enyl)phenyl]-8-(3-methylbut-2-enyl)chroman-4-one	C25H28O4	Flavonoids	3.87	393.205	457.1	[M+H]+
Glycyrrhisoflavone	C20H18O6	Isoflavonoids	3.87	355.1171	392	[M+H]+
Pomolic acid	C30H48O4	Triterpenoids	3.87	471.3486	494.8	[M-H]-
Vicenin 3	C26H28O14	Flavonoids	3.87	563.141	269.2	[M-H]-
2-amino-9-(2,7-dihydroxy-2-oxo-4a,6,7,7a-tetrahydro-4H-furo[3,2-d][1,3,2]dioxaphosphinin-6-yl)-1H-purin-6-one	C10H12N5O7P	Nucleosides	3.85	346.054	164.4	[M+H]+
7,3',4'-Trihydroxyflavone	C15H10O5	Flavonoids	3.85	269.0454	313.6	[M-H]-
Isoschaftoside	C26H28O14	Flavonoids	3.85	563.141	269.2	[M-H]-
Umbelliferone	C9H6O3	Coumarins	3.85	161.0246	304.3	[M-H]-
5,7-dihydroxy-2-(4-hydroxyphenyl)-6-[3,4,5-trihydroxy-6-(hydroxymethyl)tetrahydropyran-2-yl]-8-(3,4,5-trihydroxytetrahydropyran-2-yl)chromen-4-one	C26H28O14	Flavonoids	3.84	565.1533	265.9	[M+H]+
Salidroside	C14H20O7	Phenylethanoids (C6-C2)	3.84	299.1141	222.8	[M-H]-
Narcissin	C28H32O16	Flavonoids	3.83	625.1746	299.2	[M+H]+
7-[3-[(2R,3R,4R)-3,4-dihydroxy-4-(hydroxymethyl)tetrahydrofuran-2-yl]oxy-4,5-dihydroxy-6-(hydroxymethyl)tetrahydropyran-2-yl]oxy-3-(4-methoxyphenyl)chromen-4-one	C27H30O13	Isoflavonoids	3.83	563.1731	307.9	[M+H]+
CyclicAMP/cAMP	C10H12N5O6P	Nucleosides	3.83	328.0455	203.3	[M-H]-

(2S,3S,4S,5R,6R)-6-[(2R,3R,4S,5S,6S)-2-[[(3S,6aR,6bS,8aS,12aR,14bS)-11-carboxy-4,4,6a,6b,8a,11,14b-heptamethyl-14-oxo-2,3,4a,5,6,7,8,9,10,12,12a,14a-dodecahydro-1H-picen-3-yl]oxy]-6-carboxy-4,5-dihydroxy-tetrahydropyran-3-yl]oxy-3,4,5-trihydroxy-tetrahydropyran-2-carboxylic acid	C42H62O16	Triterpenoids	3.82	823.4078	432.7	[M+H]+
5,7-dihydroxy-2-phenyl-chroman-4-one	C15H12O4	Flavonoids	3.82	255.0665	375.5	[M-H]-
Jaranol	C17H14O6	Flavonoids	3.82	313.072	389.7	[M-H]-
Violanthin	C27H30O14	Flavonoids	3.82	577.1566	277.8	[M-H]-
(1R,2R,5S,8R,14R,15R,16S)-16-hydroxy-8-isopropenyl-1,2,14,17,17-pentamethyl-pentacyclo[11.7.0.02,10.05,9.014,18]icosane-5,15-dicarboxylic acid	C30H46O5	Triterpenoids	3.81	485.3274	455.2	[M-H]-
Epicatechin	C15H14O6	Flavonoids	3.8	291.0857	240.6	[M+H]+
Kaempferol-3-O-galactoside	C21H20O11	Flavonoids	3.8	449.1059	284.6	[M+H]+
Adenosine 3',5'-cyclic phosphate (cAMP)	C10H12N5O6P	Nucleosides	3.8	330.0592	201.7	[M+H]+
(1R,2R,4aS,6aS,6bR,10S,12aR)-1,10-dihydroxy-1,2,6a,6b,9,9,12a-heptamethyl-2,3,4,5,6,6a,7,8,8a,10,11,12,13,14b-tetradecahydropicene-4a-carboxylic acid	C30H48O4	Triterpenoids	3.8	471.3486	494.8	[M-H]-
Glucuronic acid	C6H10O7	Saccharides	3.79	175.0249	41.8	[M-H2O-H]-
Vanillic acid	C8H8O4	Phenolic acids (C6-C1)	3.79	149.0244	268.1	[M-H2O-H]-
D-Pantothenic Acid	C9H17NO5	Small peptides	3.79	218.1035	167.7	[M-H]-
(1R)-1-[(4-hydroxyphenyl)methyl]-6-methoxy-1,2,3,4-tetrahydroisoquinolin-7-ol	C17H19NO3	Tyrosine alkaloids	3.78	286.1431	239.4	[M+H]+
Naringin	C27H32O14	Flavonoids	3.78	579.1724	273.4	[M-H]-
5,7-dihydroxy-2-phenyl-8-[(2S,3R,4R,5S,6R)-3,4,5-trihydroxy-6-(hydroxymethyl)tetrahydropyran-2-yl]-6-[(2S,3R,4S,5S)-3,4,5-trihydroxytetrahydropyran-2-yl]chromen-4-one	C26H28O13	Flavonoids	3.76	547.1459	290.6	[M-H]-
Quercetin	C15H10O7	Flavonoids	3.75	285.0386	370.8	[M-H2O+H]+
Pyruvaldehyde	C3H4O2	Fatty acyls	3.74	71.0139	41.8	[M-H]-
Licoisoflavone A	C20H18O6	Isoflavonoids	3.72	355.1171	392	[M+H]+
Glabrol	C25H28O4	Flavonoids	3.72	393.2051	494.7	[M+H]+
3',5'-Cyclic guanosine monophosphate (cGMP)	C10H12N5O7P	Pseudoalkaloids	3.72	344.0402	171.6	[M-H]-
Isoquercitrin	C21H20O12	Flavonoids	3.66	463.0884	251.2	[M-H]-
Licochalcone B	C16H14O5	Flavonoids	3.62	287.09	328.2	[M+H]+
