# Supplementary material for: Microbiota-driven tryptophan metabolism and AhR triggered intestinal stem cell differentiation: mechanisms of huangqin decoction in ulcerative colitis repair
Source: Chin Med. 2026 Jan 13;21:33. doi: 10.1186/s13020-025-01302-y (PMC12797797; doi:10.1186/s13020-025-01302-y)

Fig.2 E-cad β-actin

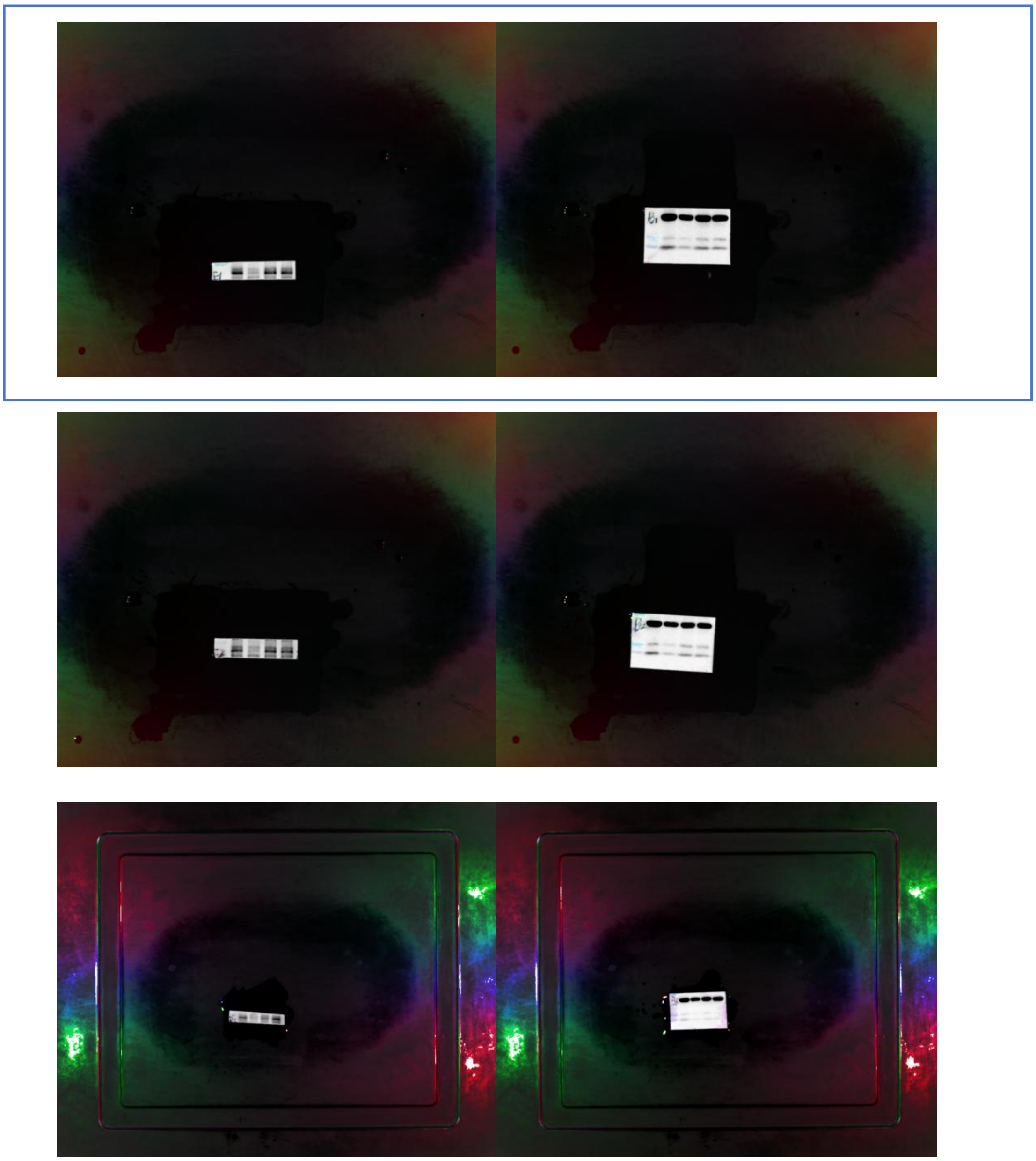

Fig.6 AhR GAPDH

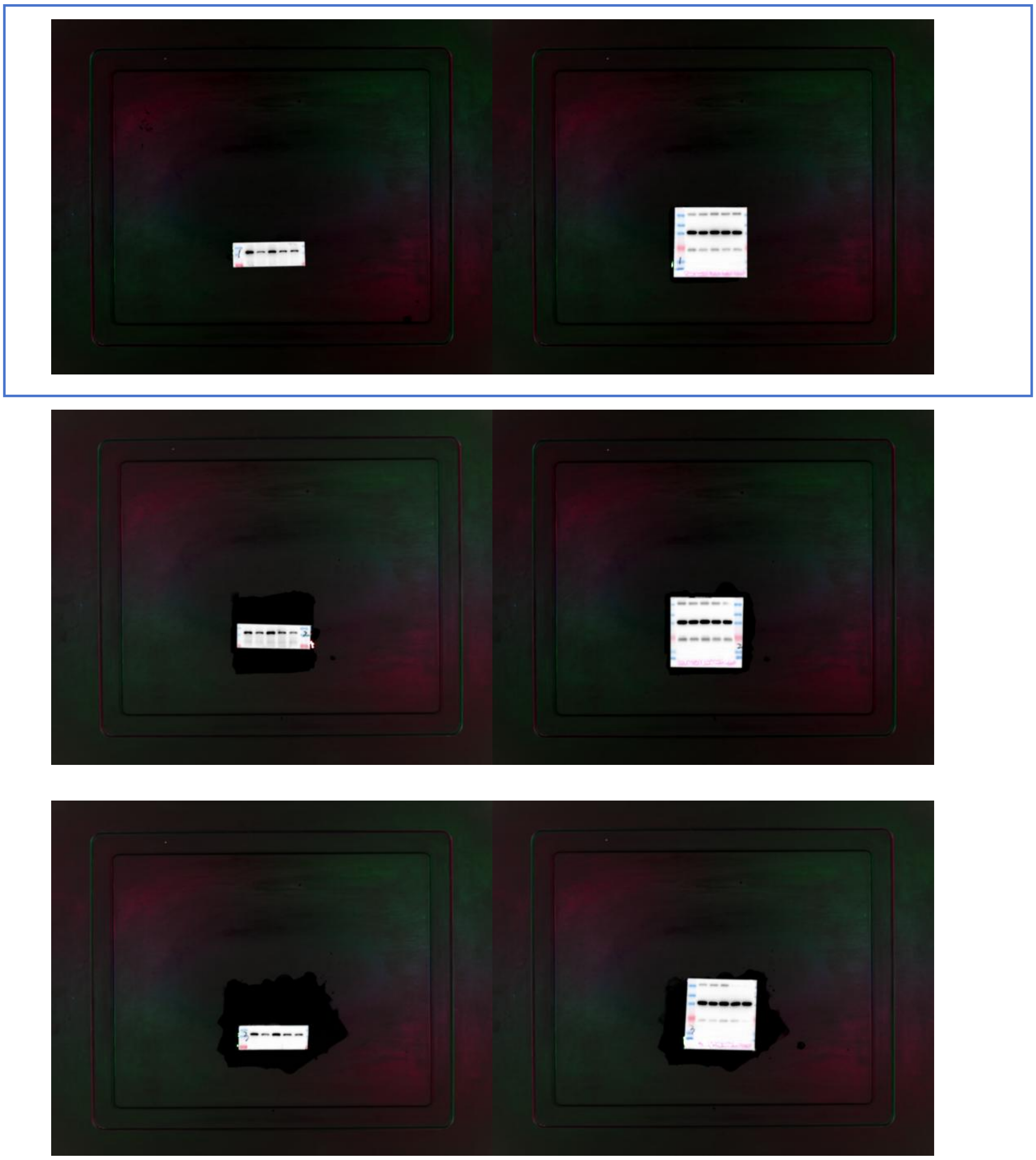

Fig.6 CYP1A1 GAPDH

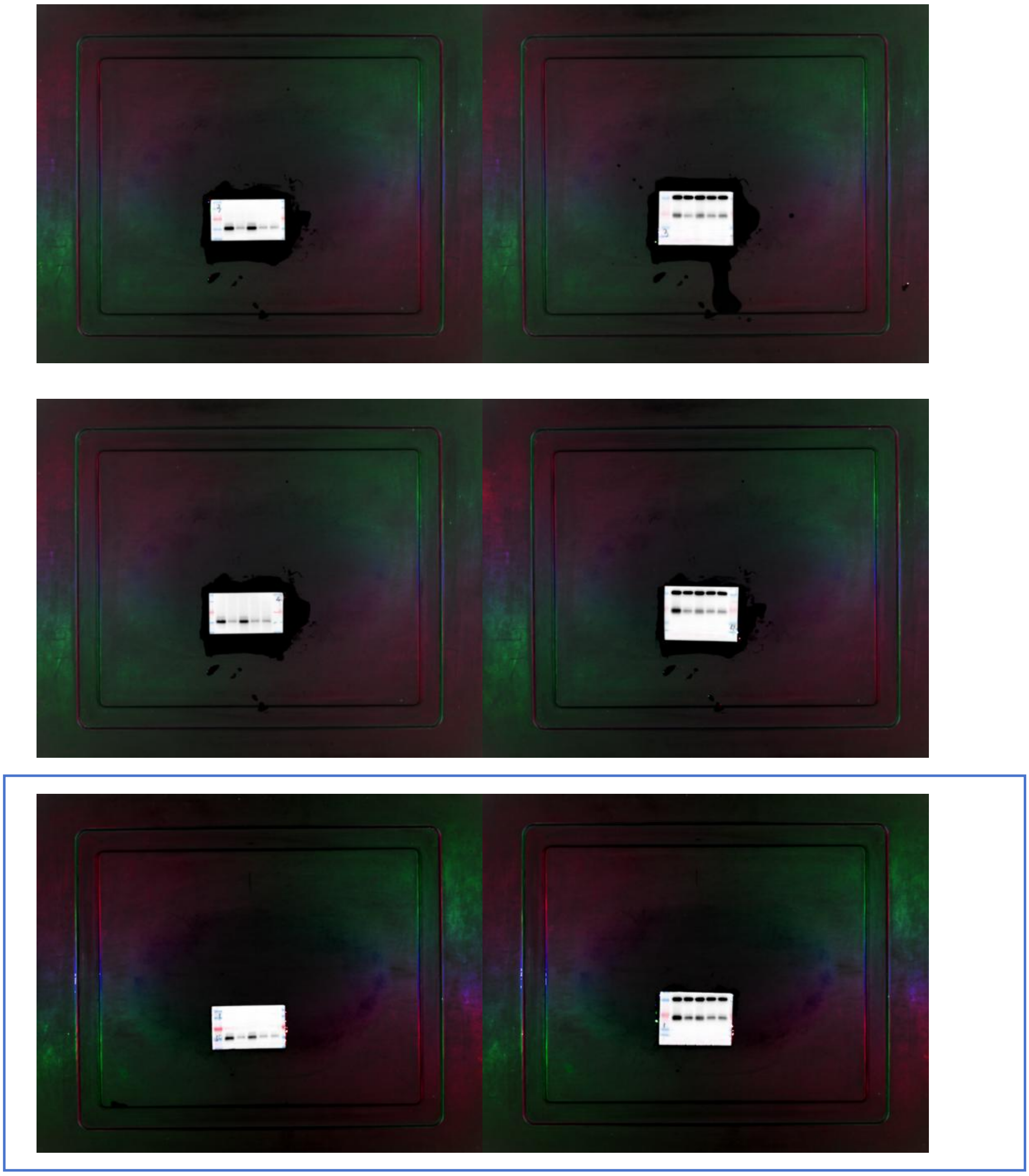

Fig.7 Lgr5 β-actin

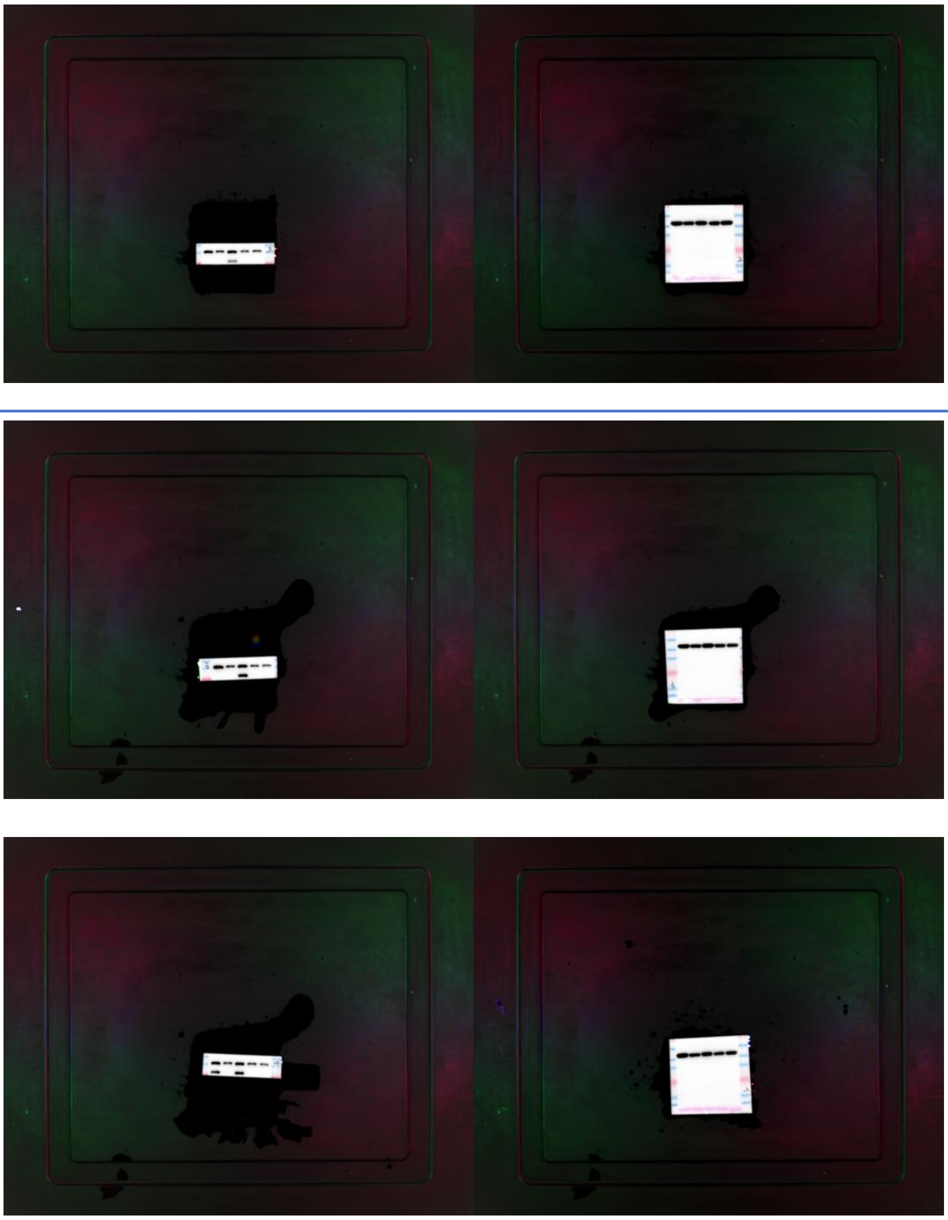

LYZ β-actin

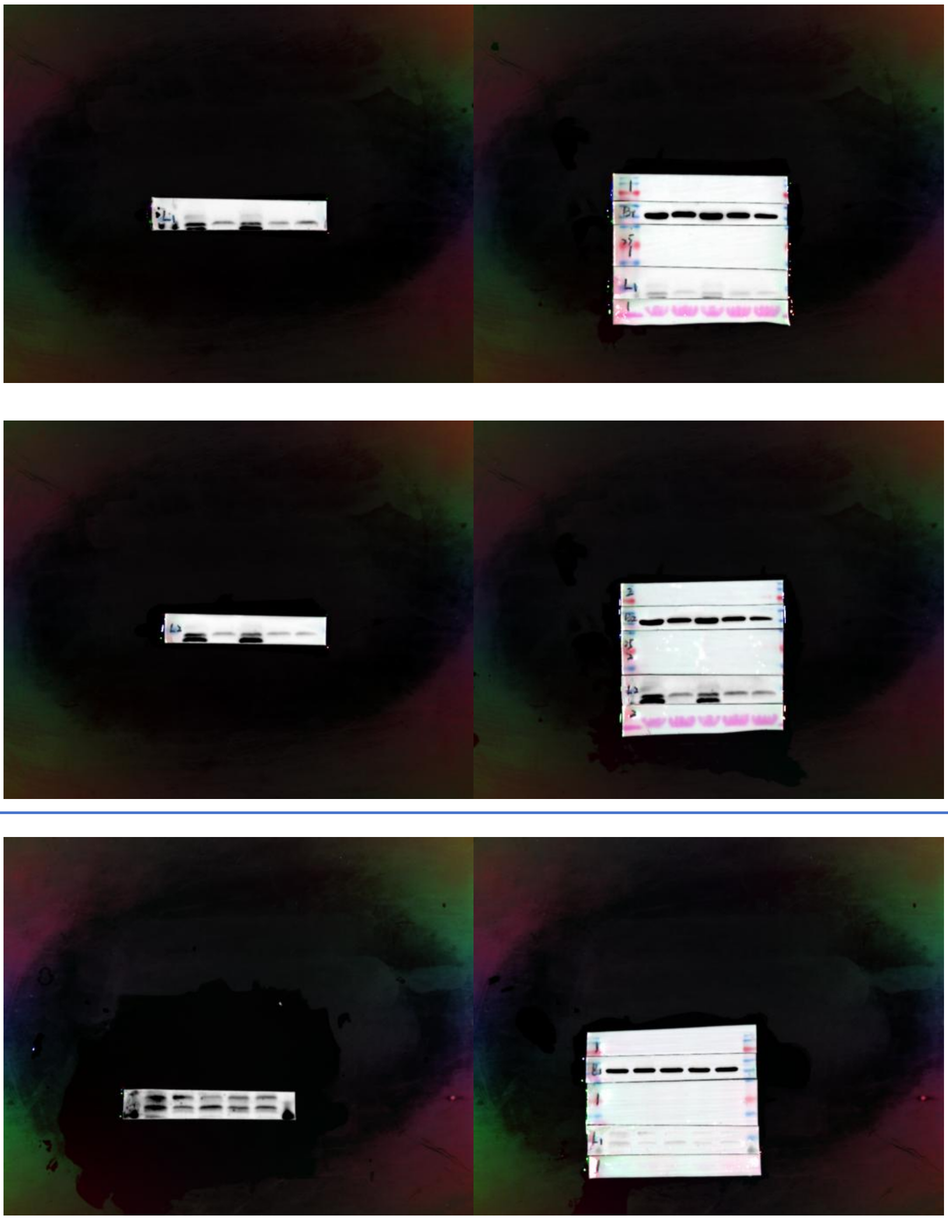

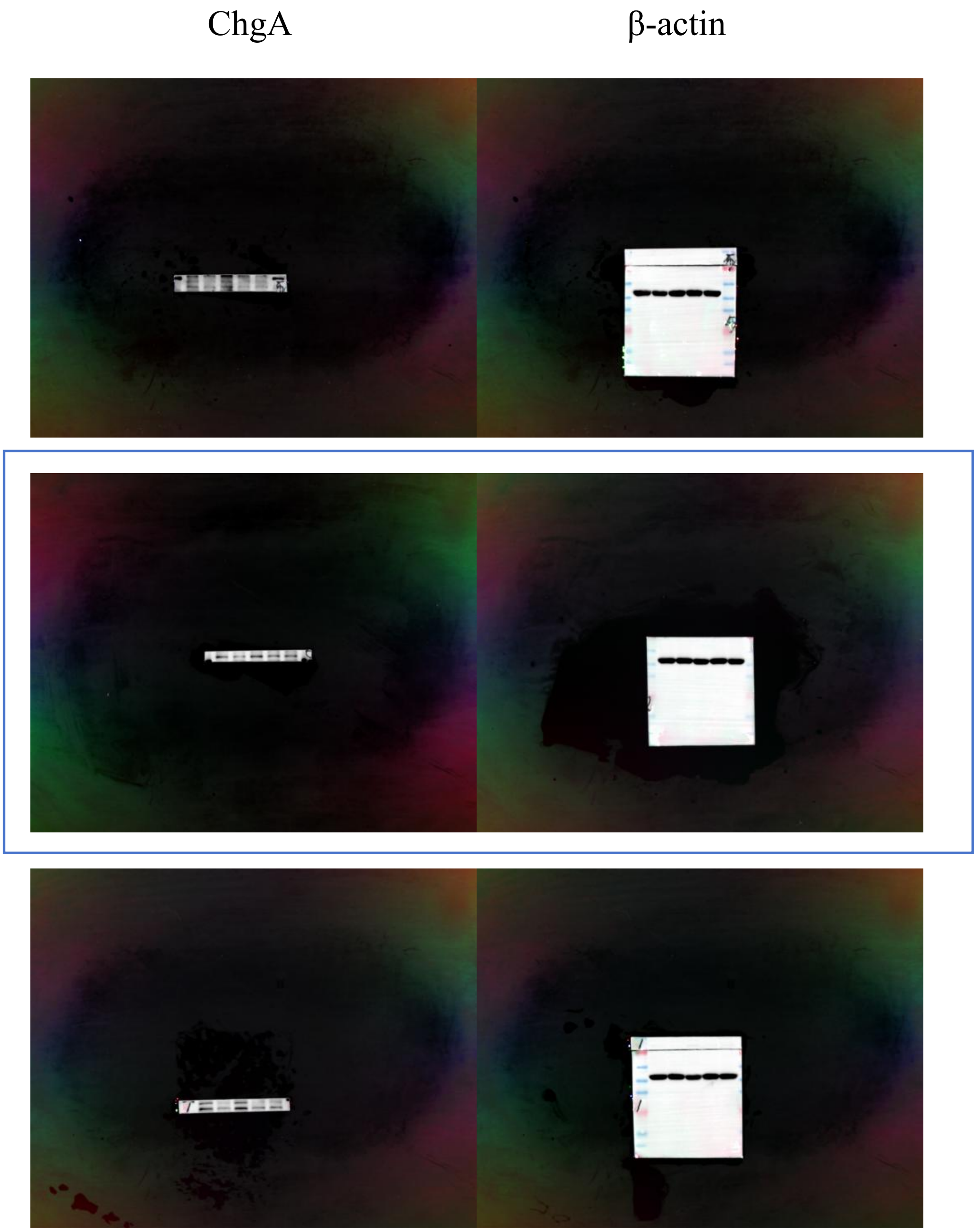
ChgA
β-actin

Fig.8 AhR GAPDH

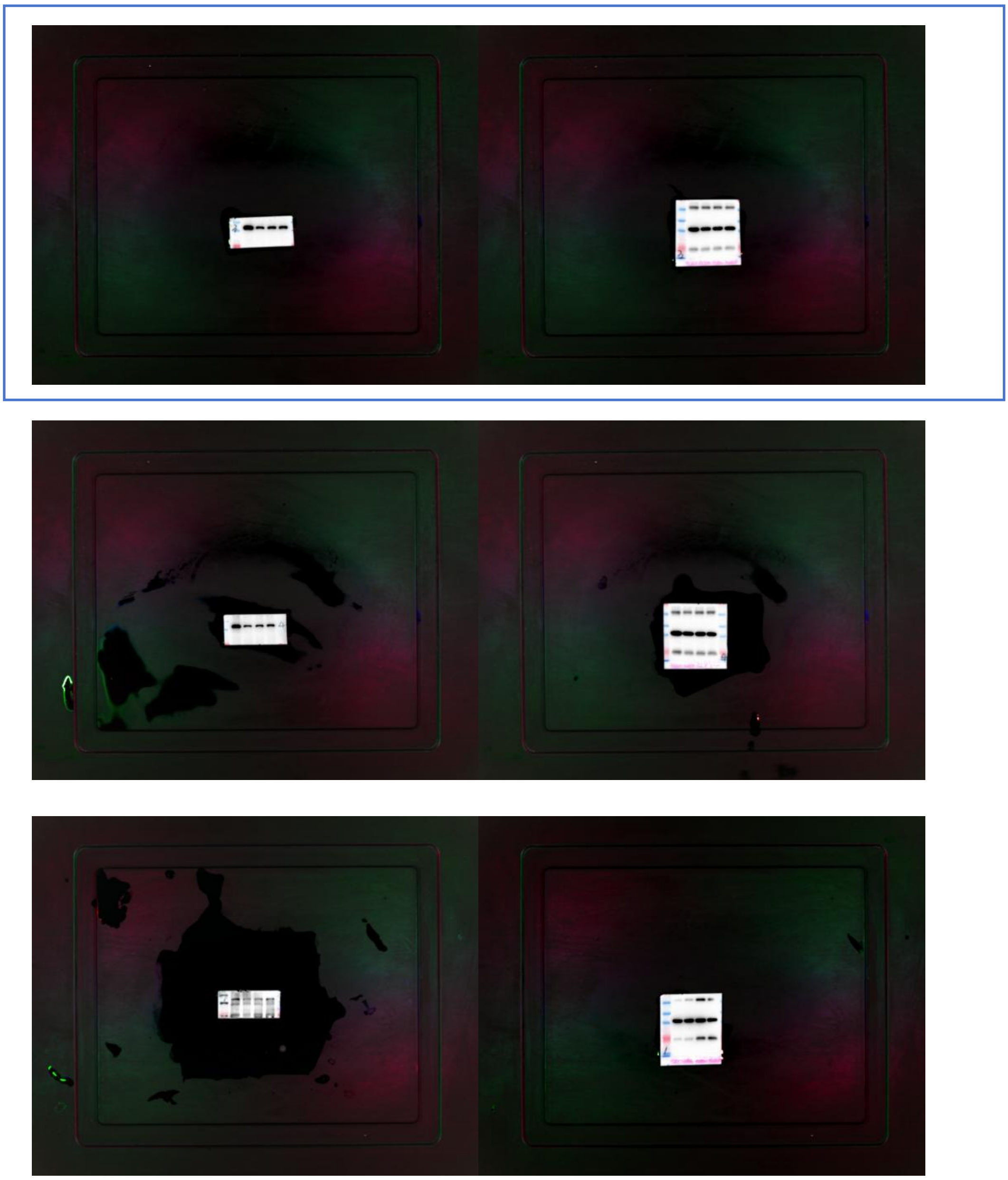

CYP1A1 GAPDH

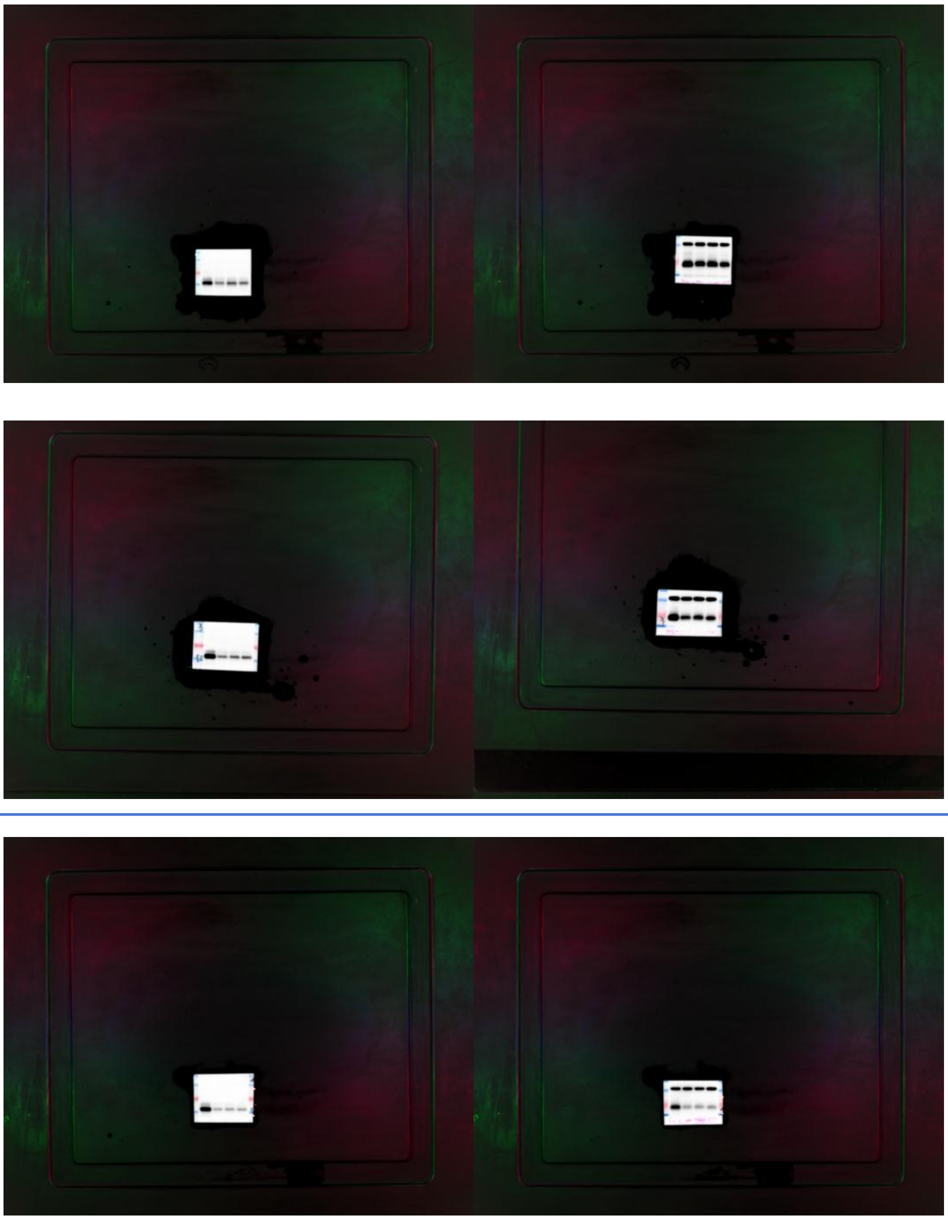

Fig.9 Lgr5 β-actin

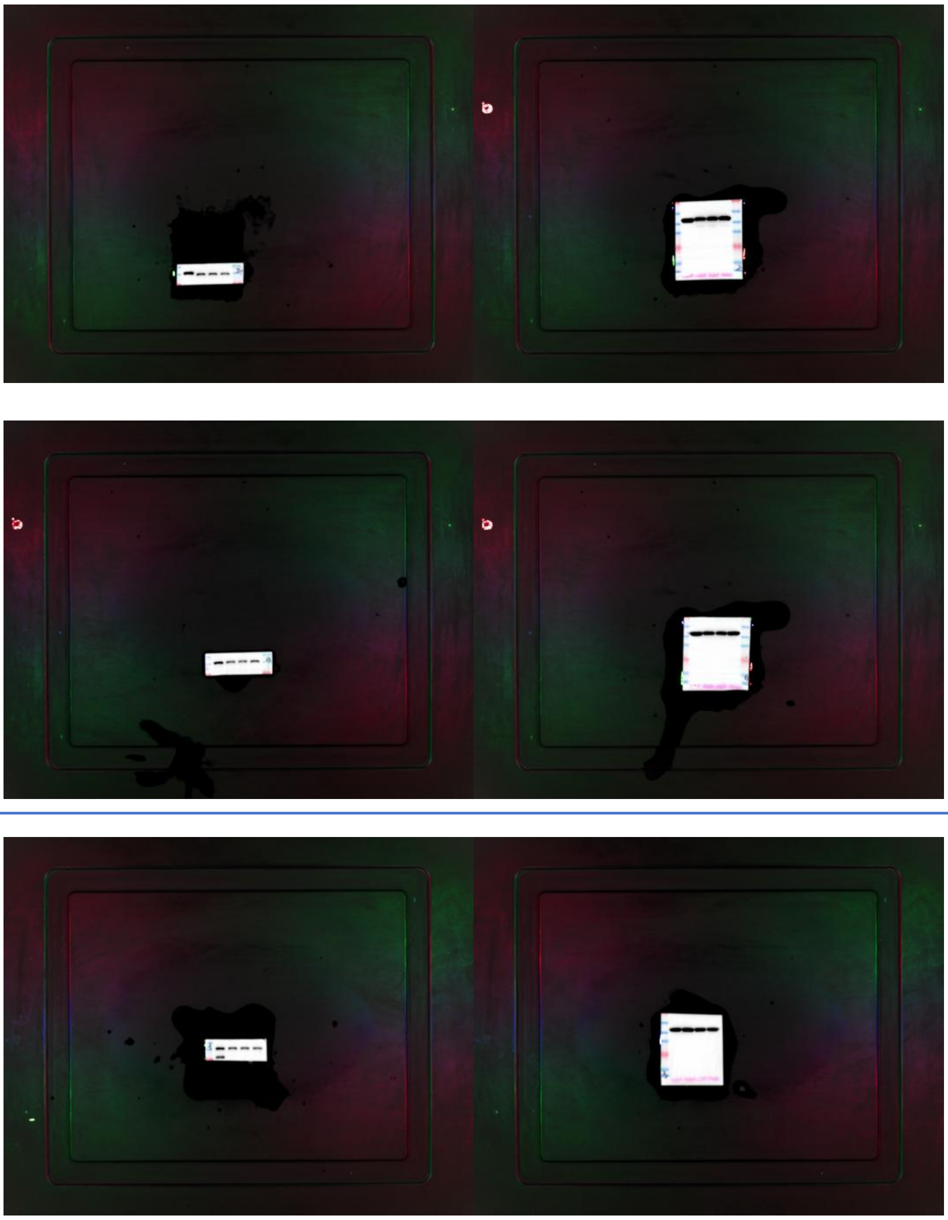

LYZ β-actin

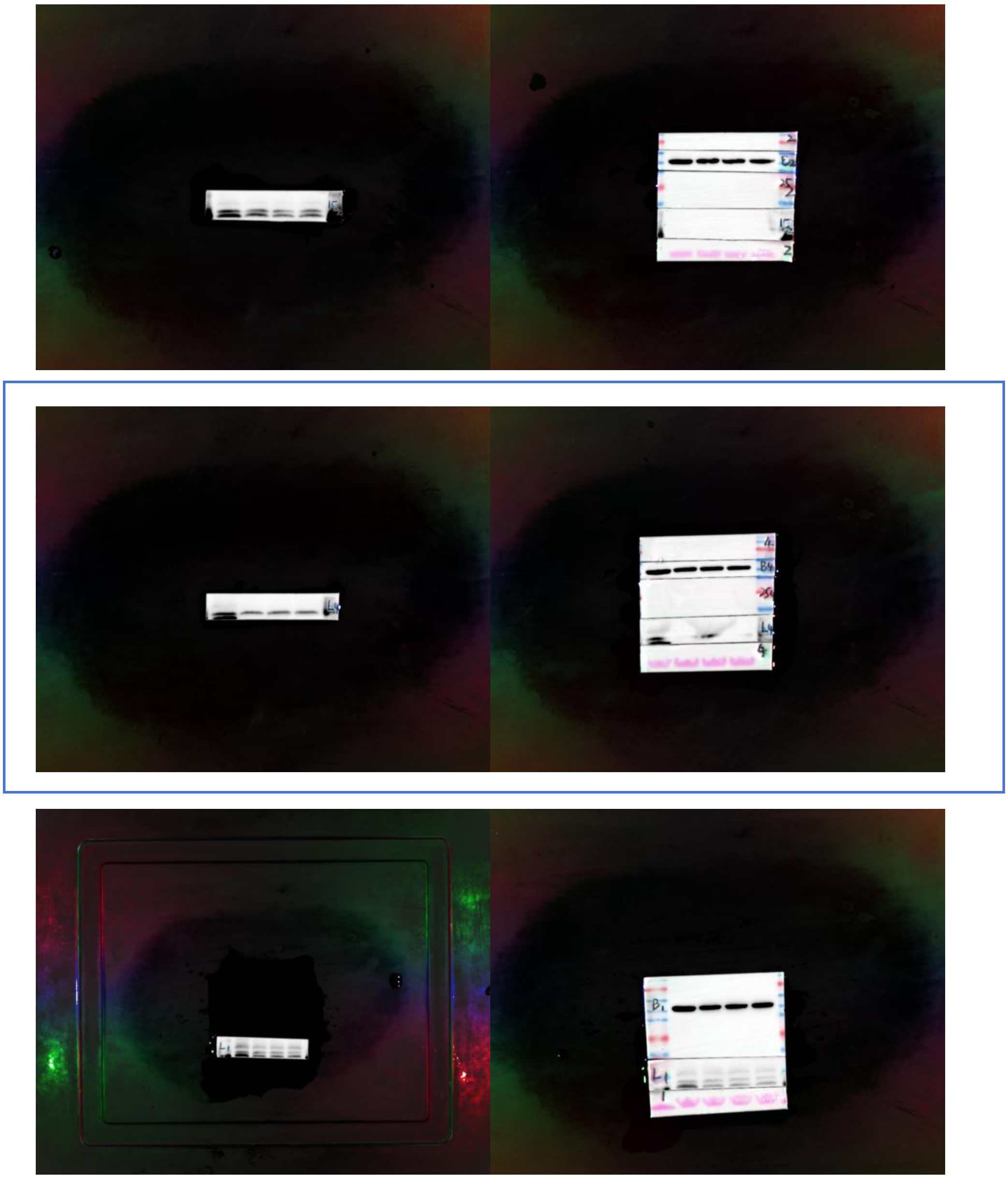

ChgA β-actin

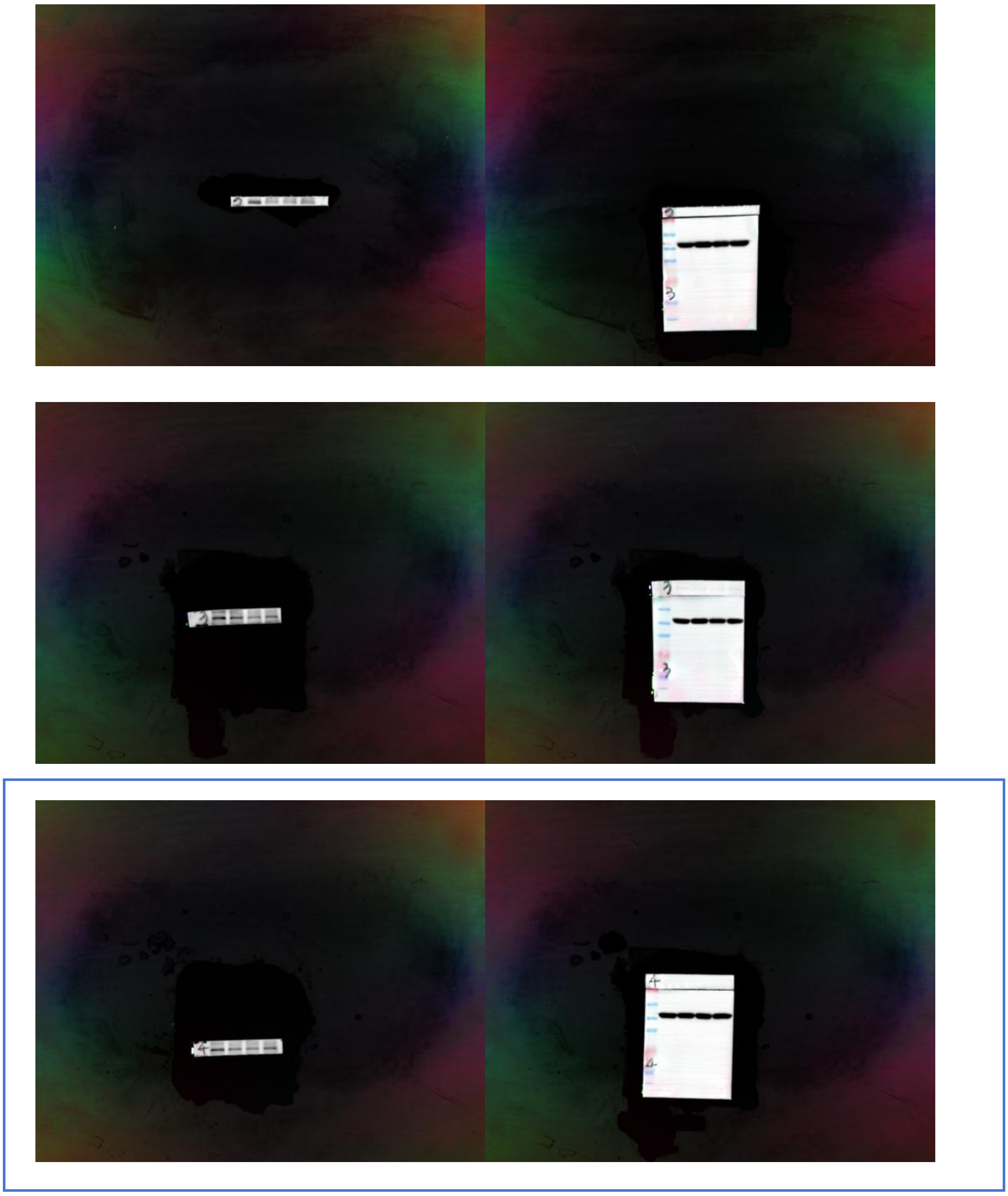

Supplement: Supplementary file 2 — Additional file 2. [file 13020_2025_1302_MOESM2_ESM.pdf]
